# Supplementary material for: A systematic benchmark of Nanopore long-read RNA sequencing for transcript-level analysis in human cell lines
Source: Nat Methods. 2025 Mar 13;22(4):801–12. doi: 10.1038/s41592-025-02623-4 (PMC11978509; doi:10.1038/s41592-025-02623-4)
Supplement: Supplementary file 1 — Supplementary Figs. 1–10, Text and Figs. 1–25, Text Tables 1 and 2, Notes 1–4 and References. [file 41592_2025_2623_MOESM1_ESM.pdf]

nature methods

Supplementary information https://doi.org/10.1038/s41592-025-02623-4

A systematic benchmark of Nanopore long-read RNA sequencing for transcript-level analysis in human cell lines

In the format provided by the authors and unedited

Content

Supplementary Figures **4**
Supplementary Fig. 1 Comparison of Nanopore RNA-Seq protocols and platforms 4
Supplementary Fig. 2 Data resource comparison 6
Supplementary Fig. 3 Comparison of Nanopore RNA-Seq protocols and platforms for spike-in samples only 6
Supplementary Fig. 4 Gene expression quantification within and across multiple protocols using multiple methods 8
Supplementary Fig. 5 Long read RNA-Seq data improves read-to-transcript assignment and transcript abundance estimation compared to short read RNA-Seq data. 10
Supplementary Fig. 6 Full-splice-match reads supported isoform analysis with long reads identifies complex transcriptional events and novel transcripts. 12
Supplementary Fig. 7 Long read RNA-Seq enables the discovery and quantification of highly repetitive genes 13
Supplementary Fig. 8 Novel transcripts identified with primary alignments and reads with only unique alignments 14
Supplementary Fig. 9 Detection and quantification of full-length fusion transcripts 15
Supplementary Fig. 10 Profiling of m6A in 7 human cell lines using direct RNA-Seq. 16
Supplementary Text **17**
1. Investigating full-length read in nanopore protocols 17
Supplementary Text Fig. 1. Percentage of full-length length reads in SG-NEx samples and publicly available data for different protocols 17
Supplementary Text Fig. 2 Average distance between observed start/ends and annotated start/ends for different RNA-seq protocols 18
Supplementary Text Fig. 3 Percentage of reads contain both 5' and 3' primers for SGNex_Hct116_cDNA_replicate3_run5 19
Supplementary Text Fig. 4 Percentage of reads covering starting and ending exons of transcripts for different RNA-seq protocols 20
2. Investigating incompatible reads observed in nanopore data 20
Supplementary Text Fig. 5 Investigation for reads incompatible to transcripts 21
3. Batch effect analysis 22
Supplementary Text Fig. 6 Heatmap showing the correlation of genes log2-transformed CPM estimates across the SG-NEx samples generated using PCR cDNA, direct cDNA, direct RNA and short read protocols when treating cell line as an batch effect 22
Supplementary Text Fig. 7 Heatmap showing the correlation of genes log2-transformed CPM estimates across the SG-NEx samples generated using PCR cDNA, direct cDNA, direct RNA and short read protocols when treating protocol as an batch effect 23
Supplementary Text Fig. 8 Heatmap showing the correlation of genes log2-transformed CPM estimates across the SG-NEx samples generated using PCR cDNA, direct cDNA, direct RNA and short read protocols using bambu for long read and RSEM for short read 23

4. Differential gene expression using long and short read RNA-seq data 24
Supplementary Text Fig. 9 Differential expression results when using long and short read 25
5. Short read simulation 25
6. Paired-end short read simulation 25
Supplementary Text Fig. 10 Paired end simulation results for one sample 26
7. Novel transcript candidates are confirmed using recent genome annotations 26
Supplementary Text Fig. 11 PCR bands observed for selected novel transcript candidates in MCF7 cell line 27
8. Statistics for novel transcript candidates 27
Supplementary Text Fig. 12 Novel transcripts detected in multiple samples, cell lines and protocols 28
9. PacBio comparison 28
Supplementary Text Fig. 13 Transcription by gene length for different sequencing protocols 28
10. Replicability using the same platform and protocol 29
Supplementary Text Fig. 14 Reproducibility between biological replicates 29
11. Pseudogenes 29
Supplementary Text Fig. 15 Percentage of reads aligned to Pseudogenes across samples when primary alignments, reads with unique alignments, or PacBio reads are used 30
12. Variation in sequencing depth for the nanopore long-read RNA-seq data 30
13. Novel transcripts are enriched in retrotransposons 31
14. Transcripts overlapping with repeat elements with different thresholds 31
Supplementary Text Fig. 16 Distribution of overlapping percentages for all transcripts and transcripts with at least 80% overlapping with repeat elements 32
Supplementary Text Fig. 17 Overview of the repeat family in expressed transcripts 32
15. Analysis of multi-mapped reads and their impact on transcript discovery 32
Supplementary Text Fig. 18 Impact of multiple-mapping reads on transcript discovery 33
16. Analysis for lowly expressed spike-in genes 33
Supplementary Text Fig. 19 Analysis of spike-in RNAs with expected CPM < 2.5. 34
17. Comparison of transcript expression using ENCODE short read RNA-seq data 34
Supplementary Text Fig. 20 ENCODE short-read RNA-Seq data and SG-NEx short-read RNA-Seq data showed similar correlations with both long-read original and fragmented data for transcript expression 35
Supplementary Text Table 1 Description of ENCODE short read RNA-Seq data with downloading links 37
18. GO enrichment analysis on genes that share or disagree major isoforms between long and short read RNA-seq data 37
Supplementary Text Fig. 21 Genes with discordant or concordant dominant isoforms between long and short reads are enriched in similar GO categories 37
19. Additional results from RT-qPCR and dPCR experiments 38
Supplementary Text Fig. 22 qPCR and dPCR results for selected candidates 39
20. Alternative isoform switching analysis with edgeR 39

21. Highly repetitive novel transcripts identified when different NDR applied and considering only retrotransposons 40
Supplementary Text Fig. 23 Novel transcripts had lower expression values, and higher repeat overlapping percentages across varying NDR thresholds 42
22. New Hek293T sample sequenced using RNA004 kit 42
Supplementary Text Fig. 24 Dorado and m6Anet performed similarly for sample generated using RNA004 43
23. Filtering potential RT and intra-priming artefacts with SQANTI3 44
24. Impact of read quality filtering on highly repetitive novel transcripts 44
Supplementary Text Fig. 25 Few novel transcripts classified as potential RTS or intra-priming (polyA percentage > 60%) artefacts 45
25. Summary of spike-in set characteristics 45
Supplementary Text Table 2 Characteristics of spike-in sets included in SG-NEx data resource 46
26. Long read enables profiling of full-length fusion transcripts 46
Supplementary Notes 47
1. Analysis of alternative isoform expression 47
2. Novel transcripts and repeat enrichment analysis 48
3. Fusion gene analysis 49
4. m6A modification analysis 50
References 53

Supplementary Figures

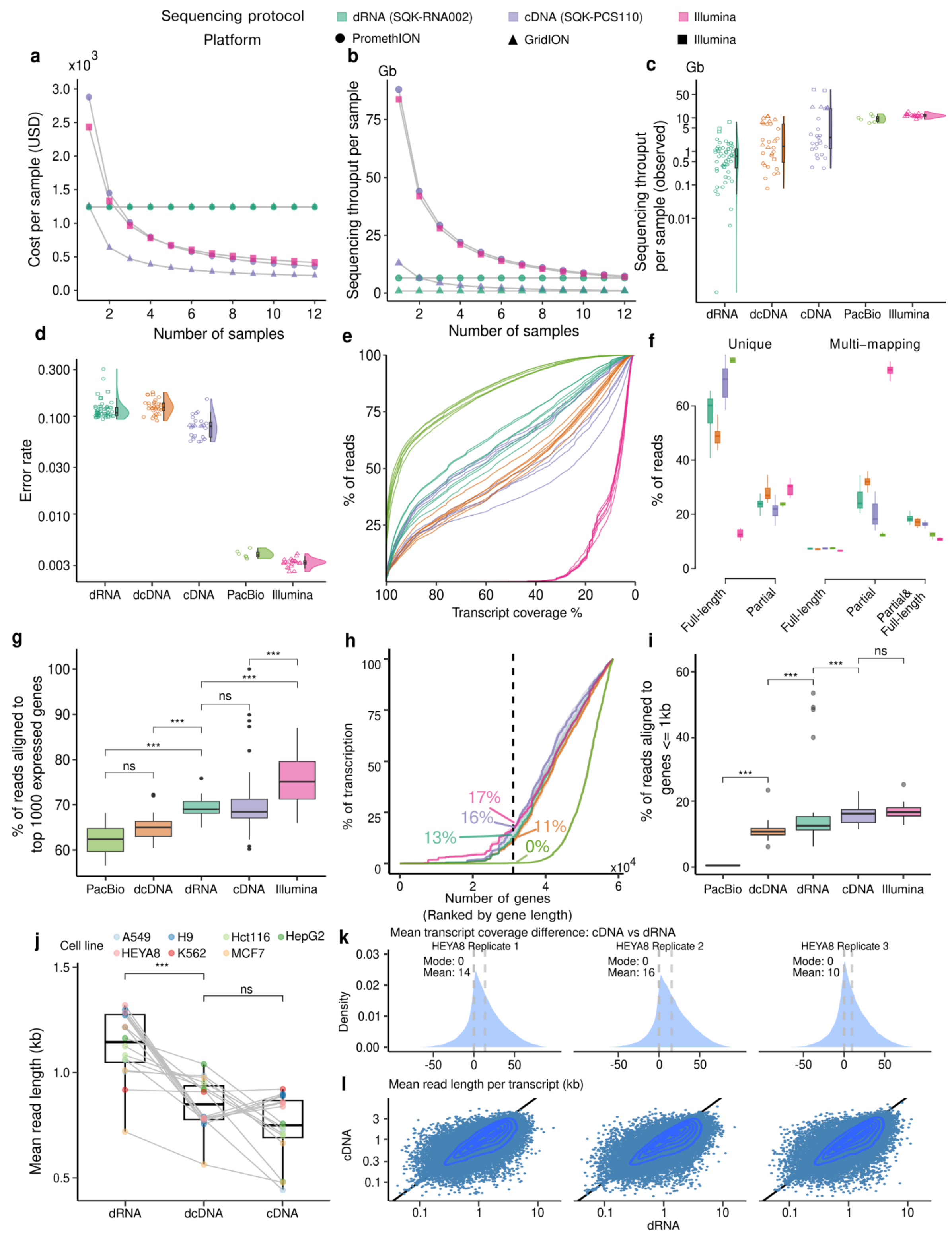

Supplementary Fig. 1 Comparison of Nanopore RNA-Seq protocols and platforms

(a) Cost of sequencing per sample when 1 to 12 samples are sequenced when using MinION/GridION, PromethION, and Illumina platforms. Cost calculations are based on charge from the service provider. **(b)**

Sequencing throughput was approximated based on the sequencing throughput of the most recent experiments multiplied by the mean read length for each of the protocols in SG-NEx samples.**(c-d)** Violin plot showing the median, upper and lower quartile, and 1.5 x interquartile ranges of the (c)cigar-mapped bases and (d) error rate of RNA (direct RNA, n=55), cDNA (direct cDNA, n=30), PCR (cDNA, n=27), PacBio IsoSeq (n=6), and Illumina (n=21) protocols, with each circle representing MinION or GridION experimental run without multiplexing, square representing PromethION and non-de-multiplexed experimental run, and triangle representing de-multiplexed experimental runs. **(e)** Cumulative distribution plots showing the proportion of reads that cover at least the given fraction of transcripts based on the annotations, for RNA (direct RNA), cDNA (direct cDNA) and PCR (cDNA) protocols. Each line representing for a cell line **(f)** Boxplots showing the median, upper and lower quartile, and 1.5 x interquartile ranges of the percentage of reads being uniquely or multi-mapped to transcripts, and whether read is full-splice-junction matched to transcript or not (full-splice-match vs partial) for all five protocols (n=55, 30, 27, 6, 21 for direct RNA, direct cDNA, cDNA, PacBio, Illumina respectively) for spliced reads originating from protein coding genes in SG-NEx samples **(g)** Boxplots showing the median, upper and lower quartile, and 1.5 x interquartile ranges of fraction of transcription the top 1000 expressed genes for direct RNA-seq (dRNA, n=55), direct cDNA (dcDNA, n=30), PCR cDNA (cDNA, n=27), PacBio long read RNA-seq (n=6) and Illumina short read RNA-seq (n=21), with two-sided t-test p-values indicated as ns for p > 0.05, and *** for p-value <=0.0001 **(h)** Cumulative distribution plots for percentage of transcription when ranking genes by gene length for direct RNA-seq (dRNA), direct cDNA (dcDNA), PCR cDNA (cDNA), PacBio long read RNA-seq and Illumina short read RNA-seq **(i)** Boxplots showing the median, upper and lower quartile, and 1.5 x interquartile ranges of fraction of transcription the top genes with gene length less than 1kb for direct RNA-seq (dRNA, n=55), direct cDNA (dcDNA, n=30), PCR cDNA (cDNA, n=27), PacBio long read RNA-seq (n=6) and Illumina short read RNA-seq (n=21), with two-sided t-test p-values indicated as ns for p > 0.05, and *** for p-value <=0.0001 **(j)** Boxplots showing the median, upper and lower quartile, and 1.5 x interquartile ranges of mean read length for all matched samples that are sequenced in all cDNA, direct cDNA and direct RNA protocols (n=16), with two-sided paired t-test p-values indicated as ns for p > 0.05, and *** for p-value <=0.0001 **(k)** Histograms of coverage differences using mean read length per transcript relative to transcript length between cDNA and direct RNA for all three HEYA8 replicates **(l)**Scatterplots of mean read length per transcript between PCR cDNA and direct RNA samples for all three HEYA8 replicates

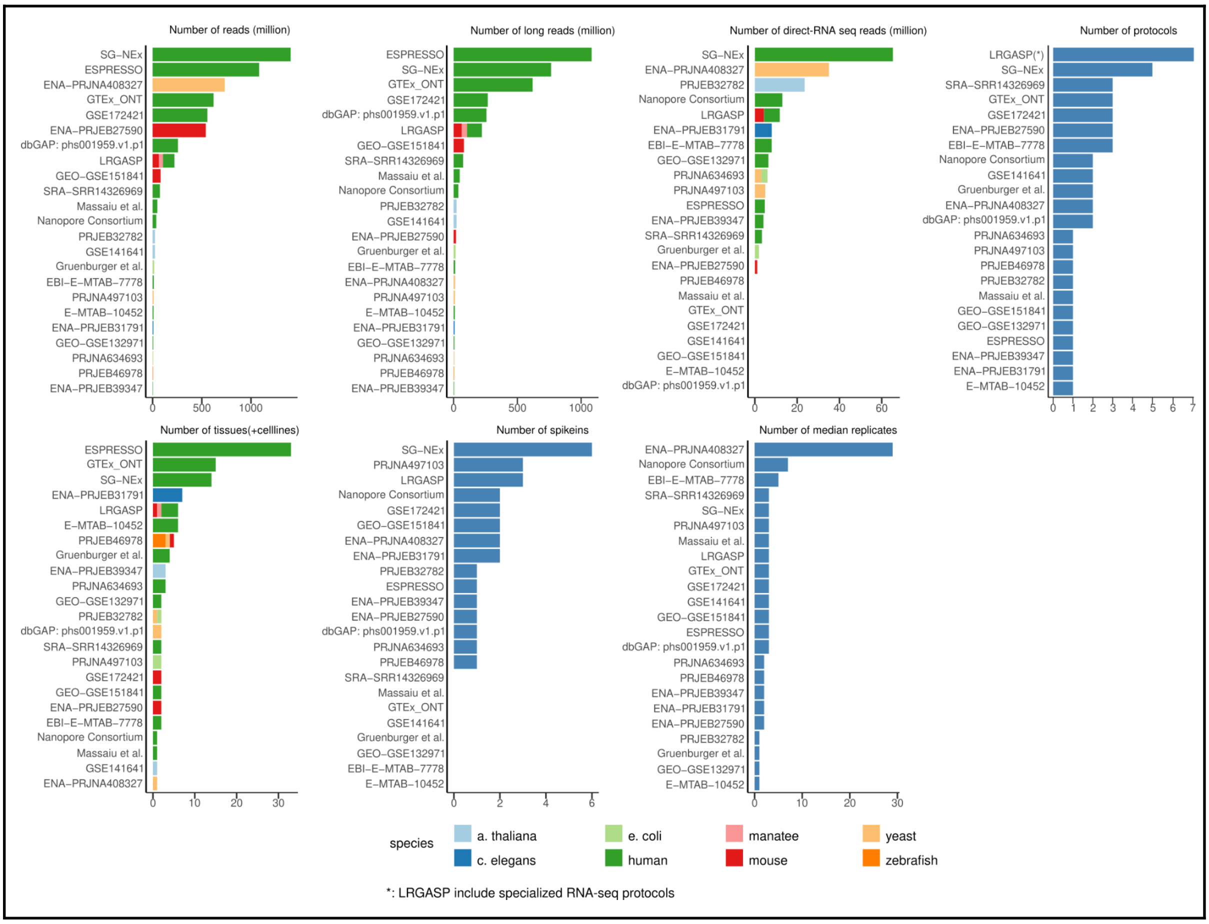

Supplementary Fig. 2 Data resource comparison
Barplots of number of reads, number of long reads, number of protocols per samples, number of cell lines (or tissues), number of spike-in sets, number of median replicates per samples, and for different long read data resources. Note for protocol comparison, LRGASP also includes specialized RNA-seq protocols.

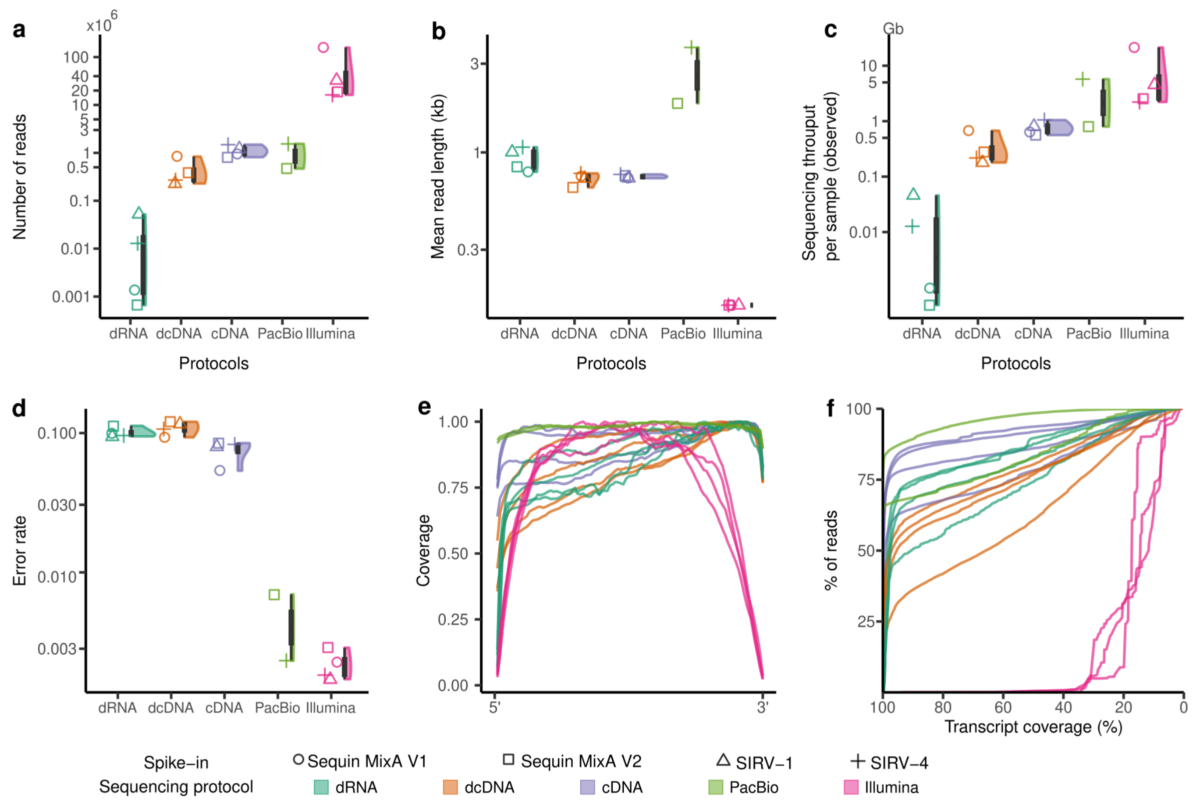

Supplementary Fig. 3 Comparison of Nanopore RNA-Seq protocols and platforms for spike-in samples only
(a-d) Violin plot showing the median, upper and lower quartile, and 1.5 x interquartile ranges of the (a) sequencing throughput, (b) mean read length, (c)cigar mapped bases and (d) error rate of RNA (direct RNA), cDNA (direct cDNA), and PCR (cDNA), PacBio IsoSeq, and Illumina (short read) protocols, for four spike-in sets: Sequin MixA V1 (circle), Sequin MixA V2 (square), SIRV-1 (triangle), and SIRV-4 (plus) (n=4) **(e)** Shown is the average coverage along the normalized transcript length for RNA (direct RNA), cDNA (direct cDNA), and PCR (cDNA), PacBio IsoSeq and Illumina protocols, with each line representing for one spike-in set **(f)** Cumulative distribution plots showing the proportion of reads that cover at least the given fraction of transcripts, for RNA (direct RNA), cDNA (direct cDNA) and PCR (cDNA) protocols, with each line representing for one spike-in set

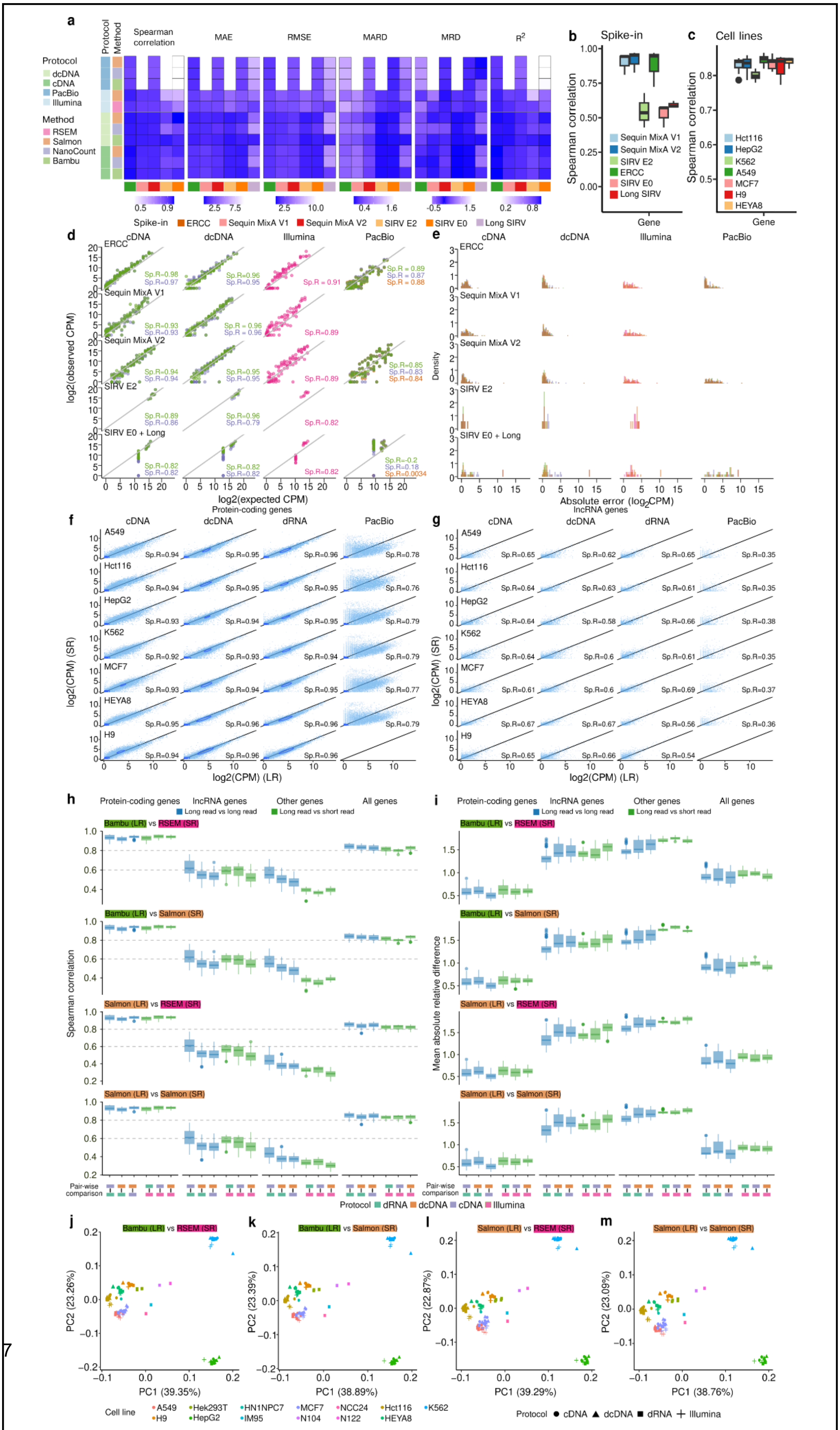

a
Protocol
Method
Spearman correlation
MAE
RMSE
MARD
MRD
R²
Protocol
dcDNA
cDNA
PacBio
Illumina
Method
RSEM
Salmon
NanoCount
Bambu
Spike-in
ERCC
Sequin MixA V1
Sequin MixA V2
SIRV E2
SIRV E0
Long SIRV
b
Spike-in
Spearman correlation
Gene
c
Cell lines
Hct116
HepG2
K562
A549
MCF7
H9
HEYA8
d
cDNA
dcDNA
Illumina
PacBio
log2(observed CPM)
log2(expected CPM)
e
Density
Absolute error (log2CPM)
Protein-coding genes
lncRNA genes
f
g
dRNA
log2(CPM) (SR)
log2(CPM) (LR)
h
i
Other genes
All genes
Long read vs long read
Long read vs short read
Bambu (LR) vs RSEM (SR)
Bambu (LR) vs Salmon (SR)
Salmon (LR) vs RSEM (SR)
Salmon (LR) vs Salmon (SR)
Spearman correlation
Mean absolute relative difference
Pair-wise comparison
Protocol
j
k
l
m
PC1 (39.35%)
PC2 (23.26%)
PC1 (38.89%)
PC2 (23.39%)
PC1 (39.29%)
PC2 (22.87%)
PC1 (38.76%)
PC2 (23.09%)
Cell line
A549
H9
Hek293T
HepG2
HN1NPC7
IM95
MCF7
N104
NCC24
N122
Hct116
HEYA8
K562

Supplementary Fig. 4 Gene expression quantification within and across multiple protocols using multiple methods
(a) Heatmaps of spearman correlation, mean absolute error (MAE), root mean squared error (RMSE), mean absolute relative difference (MARD), mean relative difference (MRD), and coefficient of determination (R2), for spike-in transcripts in ERCC, Sequin MixA V1, Sequin MixA V2, SIRV E2, SIRV E0 and Long SIRVs, when using Bambu, NanoCount and Salmon for cDNA, direct cDNA and PacBio data, and Salmon and RSEM for short read RNA-seq data **(b-c)** Boxplots showing the median, upper and lower quartile, and 1.5 x interquartile ranges of the spearman correlation between transcript expression estimates from long read samples and short read samples for both (b) spike-in (n=3) and (c) cell line (n=3) samples. Please note that the expected transcript expression is constant for all transcripts in SIRV E0, and low correlation is expected. **(d)**Scatterplots of log2-transformed CPM for spike-in genes obtained from long read direct cDNA and PCR cDNA RNA-Seq (using Salmon), and short read RNA-Seq (using Salmon with bias correction), compared against expected log2-transformed CPM for for spike-in transcripts of five different spike-in RNAs, where light blue points represent Sequin MixA version 1, SIRV E2, and dark blue points represent Sequin Mix A version 2, ERCC, and SIRV E0 + long SIRV RNAs **(e)**Histograms of absolute errors (AE) for spike-in genes in ERCC, Sequin MixA V1, Sequin MixA V2, SIRV E2, SIRV E0 and Long SIRVs, when using Bambu, NanoCount and Salmon for cDNA, direct cDNA and PacBio data, and Salmon and RSEM for short read RNA-seq data **(f-g)** Scatterplot of log2-transformed CPM for (f) protein coding genes and (g) long noncoding genes expression estimates obtained from long read PacBio, cDNA, direct cDNA and direct RNA-Seq (using Salmon) against that obtained from short read RNA-Seq (using Salmon with bias correction) in the all 7 cell lines **(h-i)**Boxplots showing the median, upper and lower quartile, and 1.5 x interquartile ranges of (h) spearman correlation and (i) mean absolute relative differences between log2-transformed CPM for protein-coding gene, long non-coding genes, rest of the genes and all genes together from replicates generated by long read protocols (LR) and short read protocol (SR) from the same cell line obtained (n=7), using Bambu and Salmon for long read data, RSEM and Salmon for short read data **(j-m)** PCA plot of sample to sample correlation for all SG-NEx samples, for cDNA (circle), direct cDNA (dcDNA, triangle), direct RNA (square), and Illumina (plus) protocols, using Bambu and Salmon for long read data, RSEM and Salmon for short read data

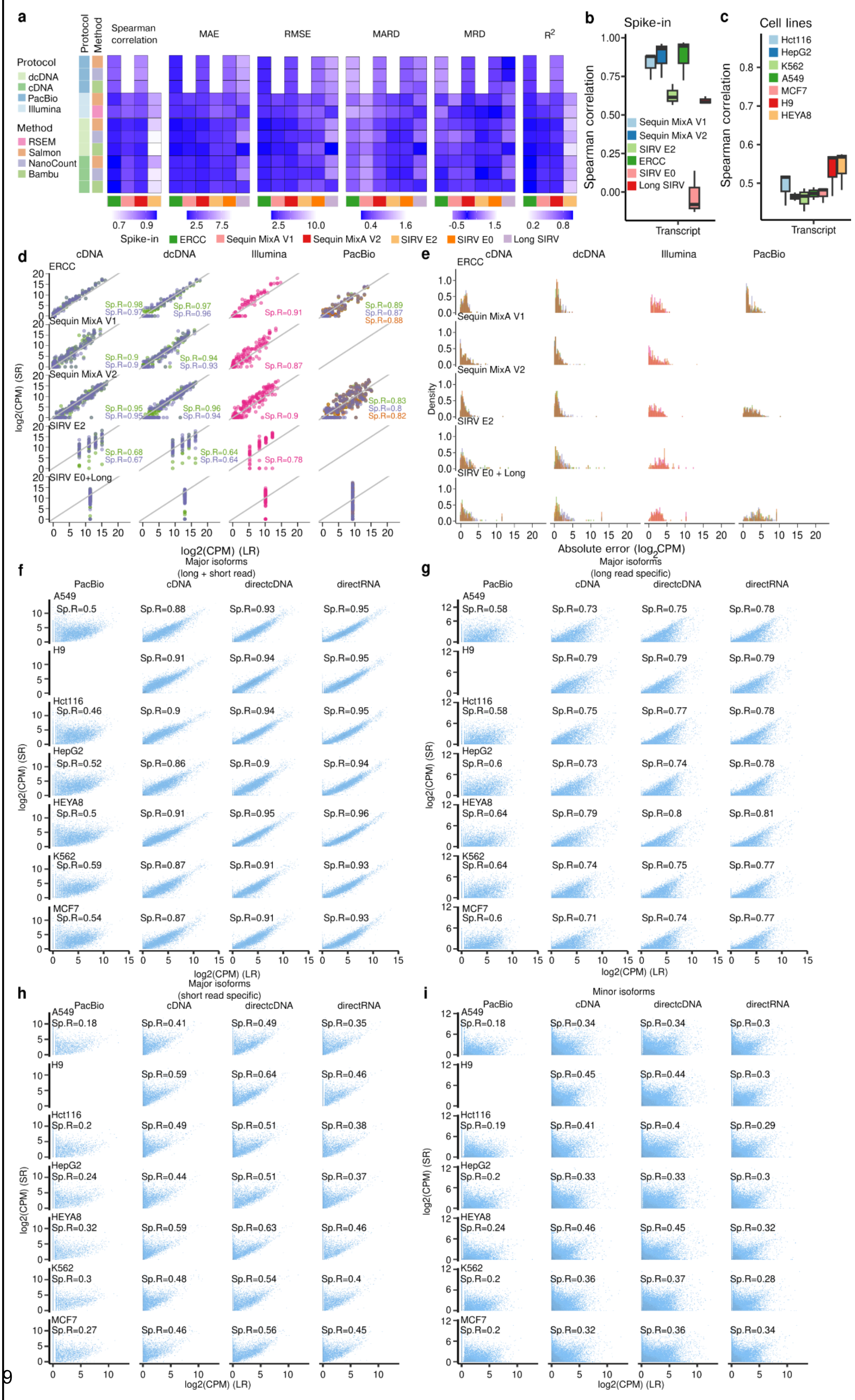
a
Protocol
Method
Protocol
dcDNA
cDNA
PacBio
Illumina
Method
RSEM
Salmon
NanoCount
Bambu
Spearman correlation
MAE
RMSE
MARD
MRD
R2
0.7 0.9
2.5 7.5
2.5 10.0
0.4 1.6
-0.5 1.5
0.2 0.8
Spike-in ERCC Sequin MixA V1 Sequin MixA V2 SIRV E2 SIRV E0 Long SIRV
b
Spike-in
Spearman correlation
1.00
0.75
0.50
0.25
0.00
Sequin MixA V1
Sequin MixA V2
SIRV E2
ERCC
SIRV E0
Long SIRV
Transcript
c
Cell lines
Spearman correlation
0.8
0.7
0.6
0.5
Hct116
HepG2
K562
A549
MCF7
H9
HEYA8
Transcript
d
cDNA
dcDNA
Illumina
PacBio
ERCC
Sp.R=0.98
Sp.R=0.97
Sp.R=0.97
Sp.R=0.96
Sp.R=0.91
Sp.R=0.89
Sp.R=0.87
Sp.R=0.88
Sequin MixA V1
Sp.R=0.9
Sp.R=0.9
Sp.R=0.94
Sp.R=0.93
Sp.R=0.87
Sequin MixA V2
Sp.R=0.95
Sp.R=0.95
Sp.R=0.96
Sp.R=0.94
Sp.R=0.9
Sp.R=0.83
Sp.R=0.8
Sp.R=0.82
SIRV E2
Sp.R=0.68
Sp.R=0.67
Sp.R=0.64
Sp.R=0.64
Sp.R=0.78
SIRV E0+Long
log2(CPM) (SR)
log2(CPM) (LR)
e
cDNA
dcDNA
Illumina
PacBio
ERCC
Sequin MixA V1
Sequin MixA V2
SIRV E2
SIRV E0 + Long
Density
Absolute error (log2CPM)
f
Major isoforms
(long + short read)
PacBio
cDNA
directcDNA
directRNA
A549
Sp.R=0.5
Sp.R=0.88
Sp.R=0.93
Sp.R=0.95
H9
Sp.R=0.91
Sp.R=0.94
Sp.R=0.95
Hct116
Sp.R=0.46
Sp.R=0.9
Sp.R=0.94
Sp.R=0.95
HepG2
Sp.R=0.52
Sp.R=0.86
Sp.R=0.9
Sp.R=0.94
HEYA8
Sp.R=0.5
Sp.R=0.91
Sp.R=0.95
Sp.R=0.96
K562
Sp.R=0.59
Sp.R=0.87
Sp.R=0.91
Sp.R=0.93
MCF7
Sp.R=0.54
Sp.R=0.87
Sp.R=0.91
Sp.R=0.93
log2(CPM) (SR)
log2(CPM) (LR)
g
Major isoforms
(long read specific)
PacBio
cDNA
directcDNA
directRNA
A549
Sp.R=0.58
Sp.R=0.73
Sp.R=0.75
Sp.R=0.78
H9
Sp.R=0.79
Sp.R=0.79
Sp.R=0.79
Hct116
Sp.R=0.58
Sp.R=0.75
Sp.R=0.77
Sp.R=0.78
HepG2
Sp.R=0.6
Sp.R=0.73
Sp.R=0.74
Sp.R=0.78
HEYA8
Sp.R=0.64
Sp.R=0.79
Sp.R=0.8
Sp.R=0.81
K562
Sp.R=0.64
Sp.R=0.74
Sp.R=0.75
Sp.R=0.77
MCF7
Sp.R=0.6
Sp.R=0.71
Sp.R=0.74
Sp.R=0.77
log2(CPM) (SR)
log2(CPM) (LR)
h
Major isoforms
(short read specific)
PacBio
cDNA
directcDNA
directRNA
A549
Sp.R=0.18
Sp.R=0.41
Sp.R=0.49
Sp.R=0.35
H9
Sp.R=0.59
Sp.R=0.64
Sp.R=0.46
Hct116
Sp.R=0.2
Sp.R=0.49
Sp.R=0.51
Sp.R=0.38
HepG2
Sp.R=0.24
Sp.R=0.44
Sp.R=0.51
Sp.R=0.37
HEYA8
Sp.R=0.32
Sp.R=0.59
Sp.R=0.63
Sp.R=0.46
K562
Sp.R=0.3
Sp.R=0.48
Sp.R=0.54
Sp.R=0.4
MCF7
Sp.R=0.27
Sp.R=0.46
Sp.R=0.56
Sp.R=0.45
log2(CPM) (SR)
log2(CPM) (LR)
i
Minor isoforms
PacBio
cDNA
directcDNA
directRNA
A549
Sp.R=0.18
Sp.R=0.34
Sp.R=0.34
Sp.R=0.3
H9
Sp.R=0.45
Sp.R=0.44
Sp.R=0.3
Hct116
Sp.R=0.19
Sp.R=0.41
Sp.R=0.4
Sp.R=0.29
HepG2
Sp.R=0.2
Sp.R=0.33
Sp.R=0.33
Sp.R=0.3
HEYA8
Sp.R=0.24
Sp.R=0.46
Sp.R=0.45
Sp.R=0.32
K562
Sp.R=0.2
Sp.R=0.36
Sp.R=0.37
Sp.R=0.28
MCF7
Sp.R=0.2
Sp.R=0.32
Sp.R=0.36
Sp.R=0.34
log2(CPM) (SR)
log2(CPM) (LR)

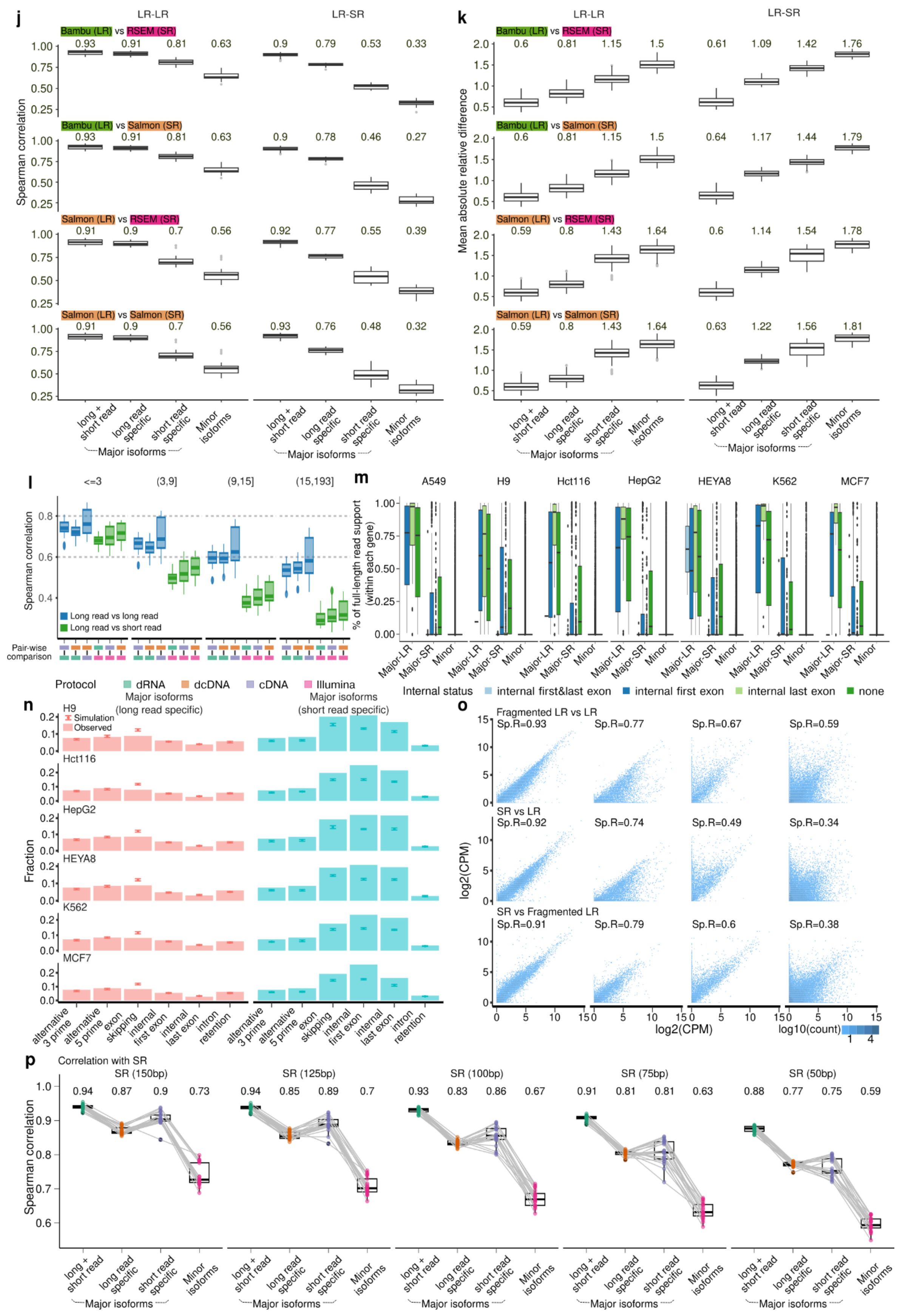

Supplementary Fig. 5 Long read RNA-Seq data improves read-to-transcript assignment and transcript abundance estimation compared to short read RNA-Seq data.

(a) Heatmaps of spearman correlation, mean absolute error (MAE), root mean squared error (RMSE), mean

absolute relative difference (MARD), mean relative difference (MRD), and coefficient of determination (R2), for spike-in transcripts in ERCC, Sequin MixA V1, Sequin MixA V2, SIRV E2, SIRV E0 and Long SIRVs, when using Bambu, NanoCount and Salmon for cDNA, direct cDNA and PacBio data, and Salmon and RSEM for short read RNA-seq data **(b-c)** Boxplots showing the median, upper and lower quartile, and 1.5 x interquartile ranges of the spearman correlation between transcript expression estimates from long read samples and short read samples for both (b) spike-in (n=3) and (c) cell line (n=3) samples. Please note that the expected transcript expression is constant for all transcripts in SIRV E0, and low correlation is expected. **(d)**Scatterplots of log2-transformed CPM for spike-in transcripts obtained from long read direct cDNA and PCR cDNA RNA-Seq (using Salmon), and short read RNA-Seq (using Salmon with bias correction), compared against expected log2-transformed CPM for for spike-in transcripts of five different spike-in RNAs, where light blue points represent Sequin MixA version 1, SIRV E2, and dark blue points represent Sequin Mix A version 2, ERCC, and SIRV E0 + long SIRV RNAs **(e)**Histograms of absolute errors (AE) for spike-in transcripts in ERCC, Sequin MixA V1, Sequin MixA V2, SIRV E2, SIRV E0 and Long SIRVs, when using Bambu, NanoCount and Salmon for cDNA, direct cDNA and PacBio data, and Salmon and RSEM for short read RNA-seq data **(f-i)** Scatterplot of log2-transformed CPM for protein coding gene (f) isoforms that major in both long and short read (Major isoforms), (g) isoforms major only in long read (Major-LR isoform), (h) isoforms major only in short read (Major-SR isoforms) and (i) isoforms that are not major isoforms (Minor isoforms) transcript expression estimates obtained from long read PacBio, cDNA, direct cDNA and direct RNA-Seq (using Salmon) against that obtained from short read RNA-Seq (using Salmon with bias correction) in the all 7 cell lines **(j-k)**Boxplots showing the median, upper and lower quartile, and 1.5 x interquartile ranges of (j) spearman correlation and (k) mean absolute relative differences between log2-transformed CPM for protein-coding gene isoforms that major in both long and short read (Major isoforms), isoforms major only in long read (Major-LR isoform), isoforms major only in short read (Major-SR isoforms) and isoforms that are not major isoforms (Minor isoforms) from replicates generated by long read protocols (LR) and short read protocol (SR) from the same cell line obtained (n=7), using Bambu and Salmon for long read data, RSEM and Salmon for short read data **(l)**Boxplots showing the median, upper and lower quartile, and 1.5 x interquartile ranges of spearman correlation between log2-transformed CPM obtained using Salmon for protein-coding gene isoforms based on the complexity quantile of gene, <=3 isoforms, 3 to 9 isoforms, 9-16 isoforms, and 16-193 isoforms, between replicates generated using different RNA sequencing protocols for each cell line (n=113, 103, 90, 90, 78, 72 for dRNA vs cDNA, dRNA vs dcDNA, cDNA vs dcDNA, dRNA vs Illumina, cDNA vs Illumina, dcDNA vs Illumina respectively), with dark blue indicating comparison between long read RNA-Seq protocols, and light blue indicating comparison between long and short read protocols. **(m)** Boxplots showing the fraction of full-splice-match read support among all aligned reads within each gene with discordant dominant isoforms between long and short read RNA-Seq data Major-LR isoform (n=2282,2636,3017,2185,2692,1679,1846 for A549, H9, Hct116, HepG2, HEYA8, K562, MCF7 respectively), Major-SR isoform (n=2282,2636,3017,2185,2692,1679,1846 for A549, H9, Hct116, HepG2, HEYA8, K562, MCF7 respectively), and Minor isoform (n=21384,25368,28579,20433,25782,15525,17487 for A549, H9, Hct116, HepG2, HEYA8, K562, MCF7 respectively), classified by their internal status for all cell lines. **(n)** Fraction of alternative events identified when comparing isoforms major only in long read (Major-LR isoform) and major only in short read (Major-SR isoforms), with background simulation distribution mean+/-SD represented by point with error bar for all other cell lines (n=20). **(o)** Scatterplots showing pairwise relationship between fragmented long read, short read RNA-seq data and long read RNA-seq data using Hct116 cell line **(p)** Boxplots showing the median, upper and lower quartiles, and 1.5 x interquartile range of the spearman correlation between log2-transformed CPM for Major isoforms, Major-LR isoforms, Major-SR isoforms, and Minor isoforms between original paired-end short read (SR) and short read-based simulated single-end 150bp, 125bp, 100bp, 75bp, 50 bp short reads (n=67).

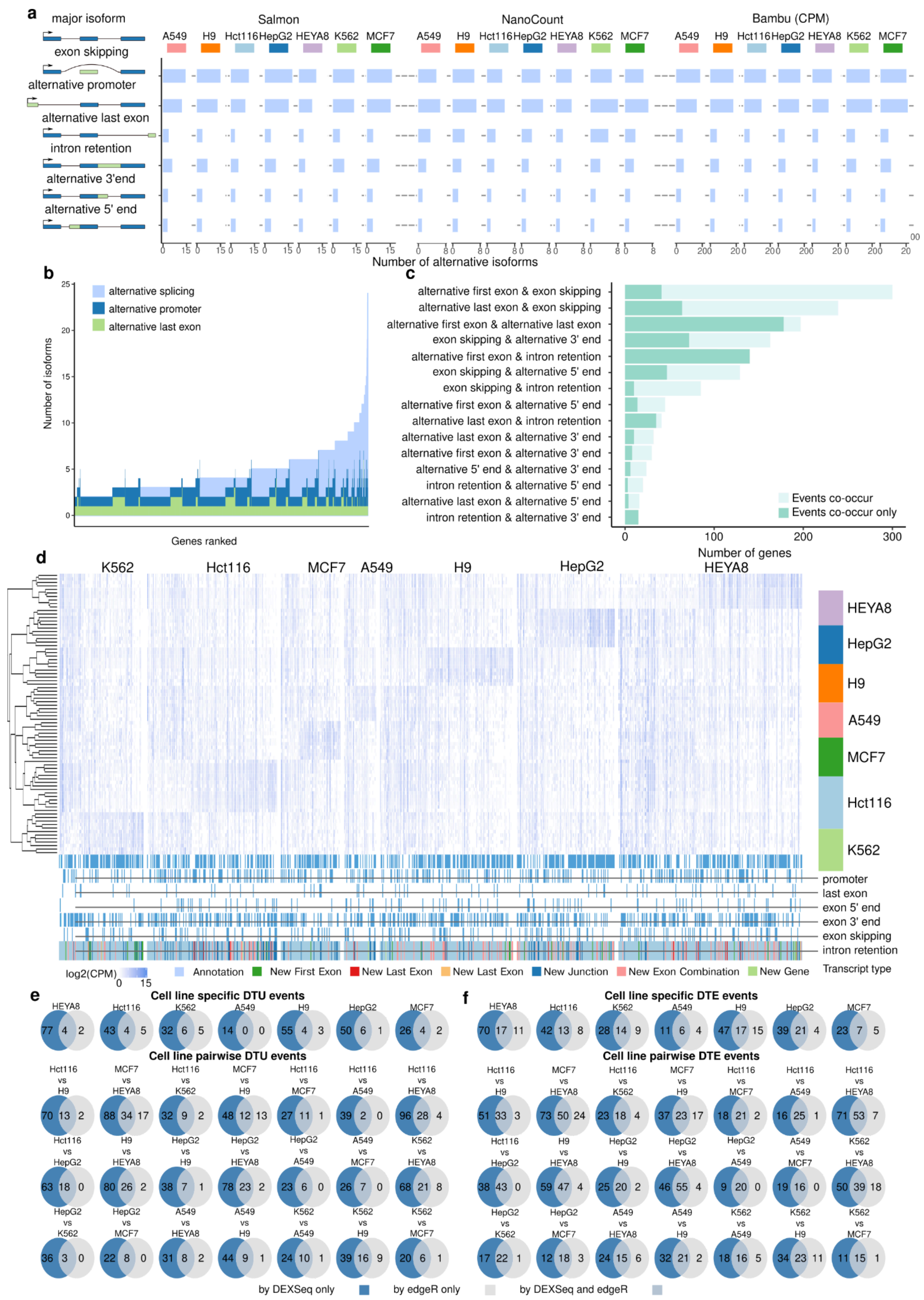

Supplementary Fig. 6 Full-splice-match reads supported isoform analysis with long reads identifies complex transcriptional events and novel transcripts.

(a) Barplots of different isoform switching type events in the 7 human cell lines using Salmon, NanoCount and Bambu without full-splice-match filtering **(b)** Barplots of genes ranked by number of isoforms expressed, with

number of promoters colored in dark blue and number of last exons colored in green. **(c)** Barplots representing co-occurence of alternative isoform events. Light blue represent number of genes with event co-occur, and darker blue represents for number of genes with event co-occur only, i.e., no independent event occurring for alternative event in this combination within each gene**(d)** Heatmap showing the expression levels of 947 isoforms showing significant isoform switching events (including minor isoforms) across the 7 human cell lines, the type of events associated with the isoform is indicated at the bottom. Expression is shown for the cell-type-specific isoforms.

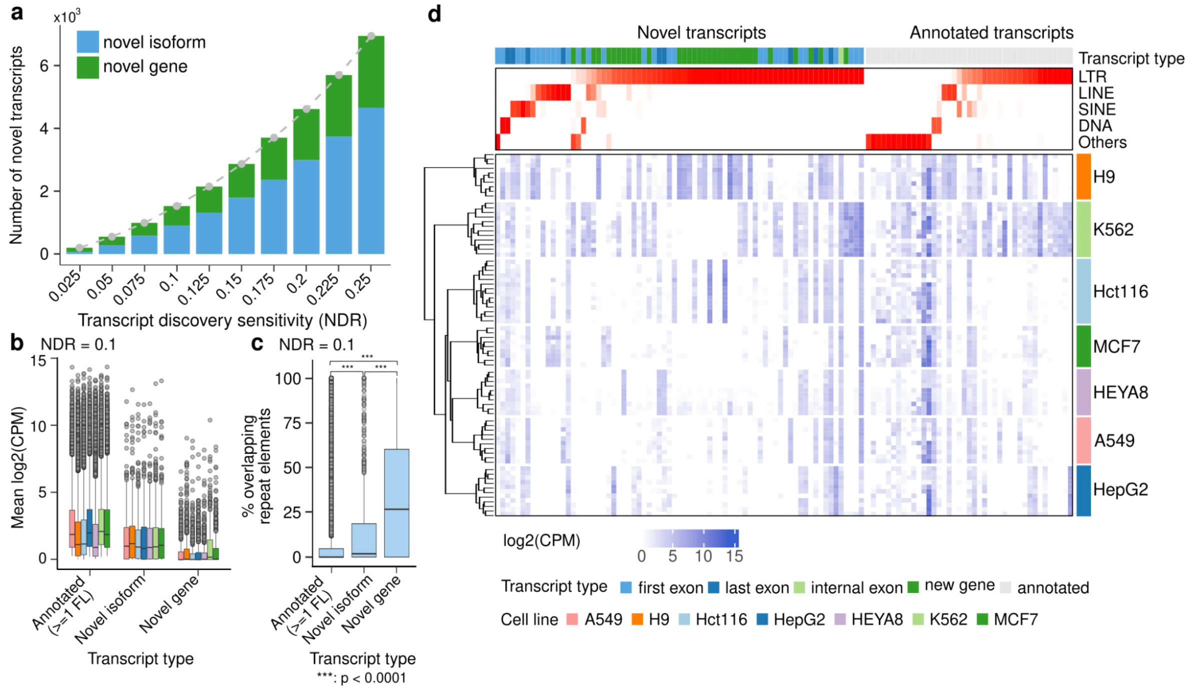

Supplementary Fig. 7 Long read RNA-Seq enables the discovery and quantification of highly repetitive genes

(a) Barplots of all novel isoforms and novel gene isoforms identified with varying transcript discovery sensitivity (NDR) from 0.025 to 0.25, with a step of 0.025. Dotted grey line represents the total number of novel transcripts. **(b)** Boxplots showing the median, upper and lower quartile, and 1.5 x interquartile ranges of the mean CPM for annotated transcripts with at least 1 full-splice-match read support across samples (Annotated >=1 FL, n=35153,51858,45733,34588,44897,33692,37643 for A549, H9, Hct116, HepG2, HEYA8, K562, MCF7 respectively), all novel isoforms (n=908), and all novel gene isoforms (n=617), for 7 cell lines. **(c)** Boxplots showing the median, upper and lower quartile, and 1.5 x interquartile ranges of the percentage of exon sequence overlapping with repeat elements for annotated transcripts with at least 1 full-splice-match read support across samples (Annotated >=1 FL, n=74455), all novel isoforms (n=908), and all novel gene isoforms (n=617), with Bonferroni corrected two-sided Mann-Whitney U test p-values reported for the pairwise median differences, asterisks indicating p < 0.0001 **(d)** Overview of the repeat family (top) and expression levels across samples in the 7 human cell lines (bottom) for transcripts with at least 80% overlap with repeat elements and expressed with >= 20 CPM in any sample, categorised into major repeat elements families (LTR, LINE, SINE, and DNA) and others

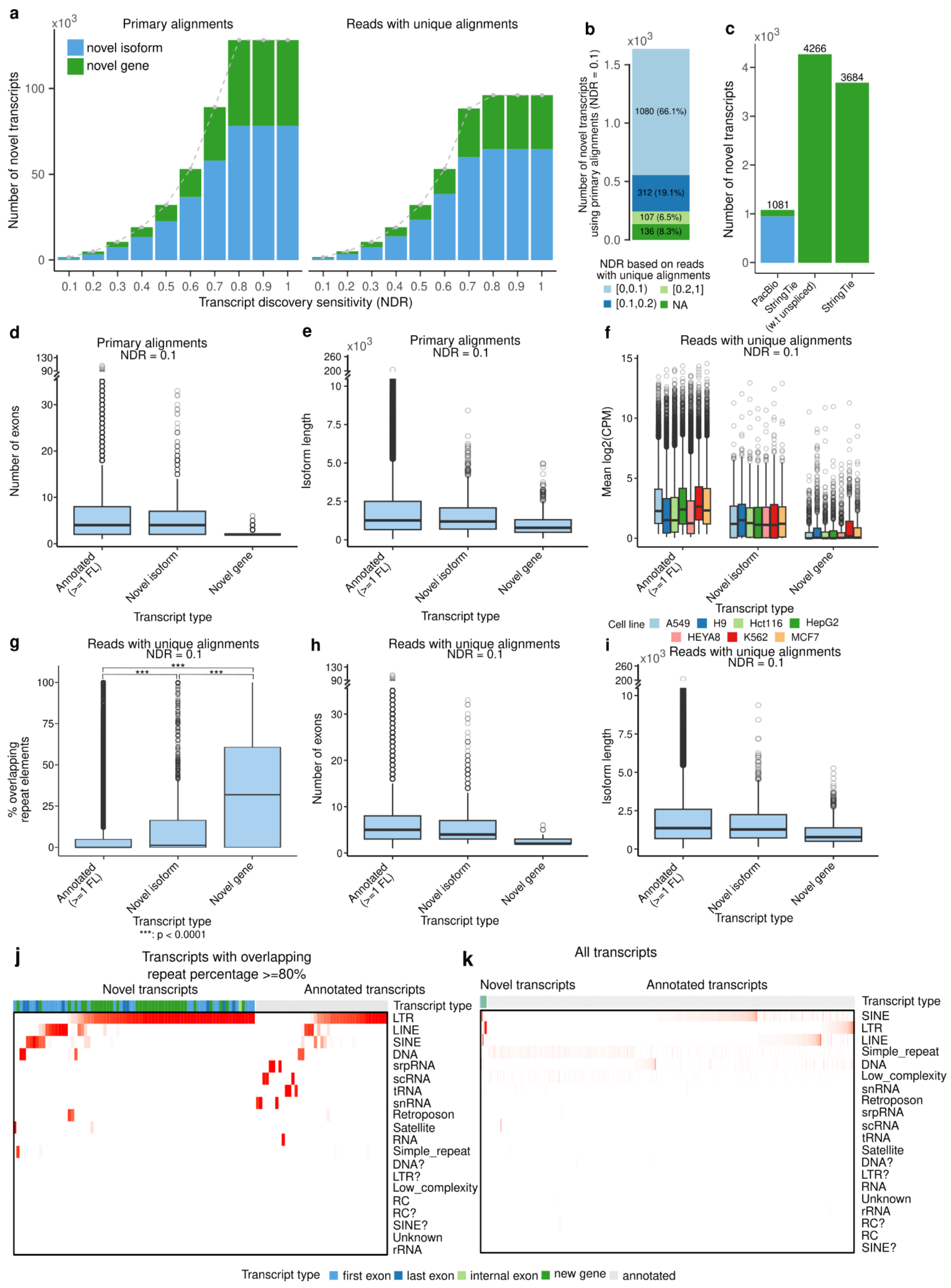

Supplementary Fig. 8 Novel transcripts identified with primary alignments and reads with only unique alignments

(a) Barplots presenting the number of all novel transcripts identified when primary alignments of all reads are used vs reads with unique alignments are used with varying transcript discovery threshold ranging from 0.1 to 1, at an increasing step of 0.1 Blue bars represent the number of novel isoforms of annotated genes discovered, green represents the number of novel isoforms of novel genes discovered. The grey points represent the total number of novel transcripts.**(b)** Barplots representing the predicted NDR when using reads

with unique alignments for all novel transcripts identified when using primary alignments with an NDR threshold of 0.1: light blue represents a predicted NDR < 0.1, blue represents a predicted NDR of >= 0.1 and < 0.2, light green represents a predicted NDR >=0.2, and green represents not identified **(c)** Barplots presenting the number of all novel transcripts identified when using PacBio data, StringTie2 method with and without unspliced novel transcripts included **(d-e)** Boxplots showing the median, upper and lower quartile, and 1.5 x interquartile ranges of (d) number of exons and (e) isoform length for annotated transcripts with at least 1 full-splice-match read support across samples (Annotated >=1 FL, n=74455), all novel isoforms (n=908), and all novel gene isoforms (n=617) using primary alignments of all reads **(f-i)** Boxplots showing the median, upper and lower quartile, and 1.5 x interquartile ranges of (f) mean log2-transformed CPM, (g) percentage overlapping with repeat elements, with Bonferroni corrected two-sided Mann-Whitney U test p-values reported for the pairwise median differences, asterisks indicating p < 0.0001, (h) number of exons and (i) isoform length for annotated transcripts with at least 1 full-splice-match read support across samples (Annotated >=1 FL, n=32040,46706,42064,31692,40297,30452,34143 for A549, H9, Hct116, HepG2, HEYA8, K562, MCF7 respectively, and 68444 across 7 cell lines), all novel isoforms (n=1030), and all novel gene isoforms (n=651) using reads with unique alignments

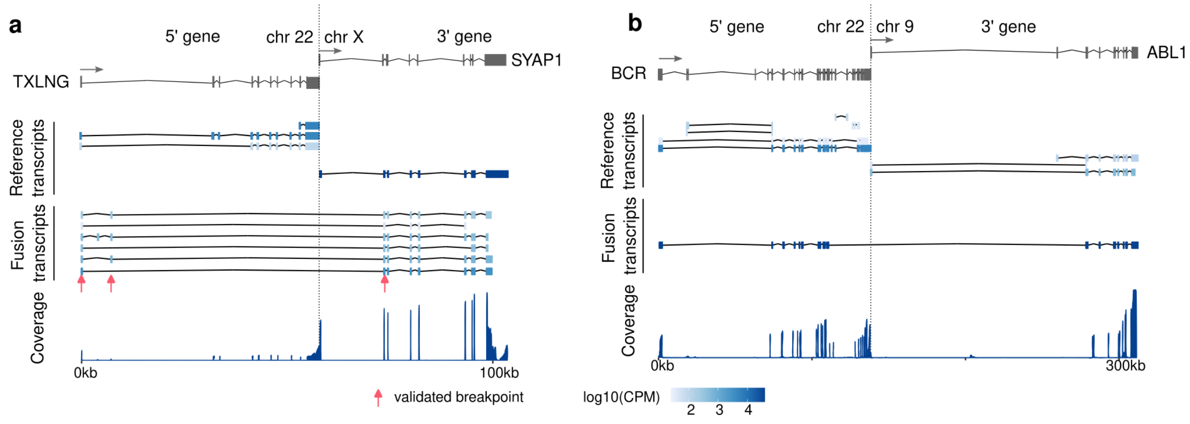

Supplementary Fig. 9 Detection and quantification of full-length fusion transcripts

(a) The isoform annotations for *TXLNG*, *SYAP1*, and the *TXLNG-SYAP1* fusion gene with color shading indicating the level of full-splice-match read support (top), and coverage plot in the region (bottom) in the MCF7 cell line. The *TXLNG-SYAP1* fusion gene shows alternative splicing patterns not observed in the 3' and 5' genes. Arrows indicate primer design for validation experiments. **(b)** The isoform annotations for *BCR*, *ABL1*, and the *BCR-ABL1* fusion genes, with color shading indicating the level of full-splice-match read support (top), and read coverage plot (bottom) in the K562 cell line. Novel transcripts for 5' and 3' genes and similar fusion transcripts are removed for simplified visualisation.

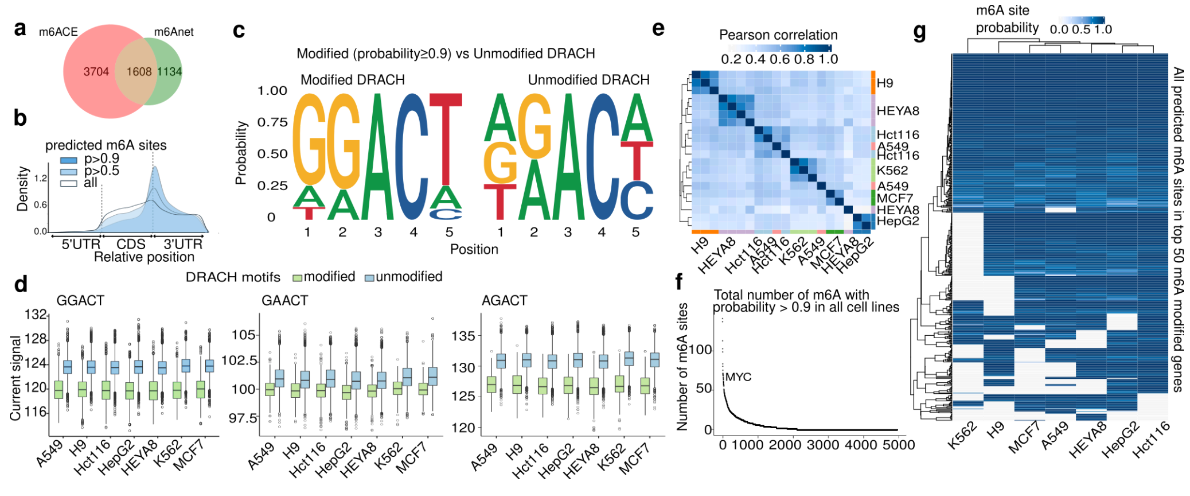

Supplementary Fig. 10 Profiling of m6A in 7 human cell lines using direct RNA-Seq.
(a) Venn diagram showing the number of overlapping m6A sites identified by m6ACE-seq and m6Anet **(b)** Metagene plot showing the frequency of m6A sites identified from direct RNA-Seq data at relative positions within the 5'UTR, CDS, and 3'UTR of a transcript. The grey line ('all') indicates the expected distribution based on all positions which are tested. **(c)** Nucleotide frequency at the five positions of all k-mers from candidate m6A positions identified by m6Anet (left) and from positions which were not identified to be modified (right). Only DRACH motifs were scanned for m6A. **(d)** Boxplot showing the median, upper and lower quartiles, and 1.5 x interquartile ranges of the current signals of modified and unmodified GGACT (n=11756 vs 45323), GAACT (n=5356 vs 56654) and AGACT (n=4036 vs 52544) positions across the seven cell lines. Outliers are shown as points. **(e)** Heatmap showing the Pearson correlation across sample replicates from the seven cell lines. **(f)** Genes ranked by the total number of m6A sites across cell lines. *MYC* ranks 40. **(g)** Heatmap of m6Anet-predicted probability of m6A sites found in top 50 genes with the highest total number of m6A sites across cell lines.

Supplementary Text

1. Investigating full-length read in nanopore protocols

(1) Reads corresponding to full length transcripts

Since transcription start and end sites are often not precisely annotated [1,2], we define any read that covers all splice junctions of a transcript as "full length" (corresponding to the commonly used definition of Full Splice Match/FSM [2,3] (see paragraph below for additional analysis). Using this definition, we observe that the long read RNA-Seq data on average contains 29.2% (direct cDNA), 49.9% (direct RNA), 51.3% PCR-cDNA, and 62.1% (PacBio IsoSeq) full length reads. If we only consider the fraction of full length reads among all spliced reads at protein coding genes, this fraction further increases to 53.2% (direct cDNA), 64.8% (direct RNA), 71.3% (PCR-cDNA), and 76.7% (PacBio IsoSeq) (see Supplementary Fig. 1f). We compared these results against a recent study using the direct RNA-seq protocol for samples with different quality values (RIN) [4]. We find that these samples provide comparable estimates of full length read counts, with higher quality samples having more full length reads (45.4% for all reads, and 59.8% for spliced reads at protein coding genes) (Supplementary Text Fig. 1)

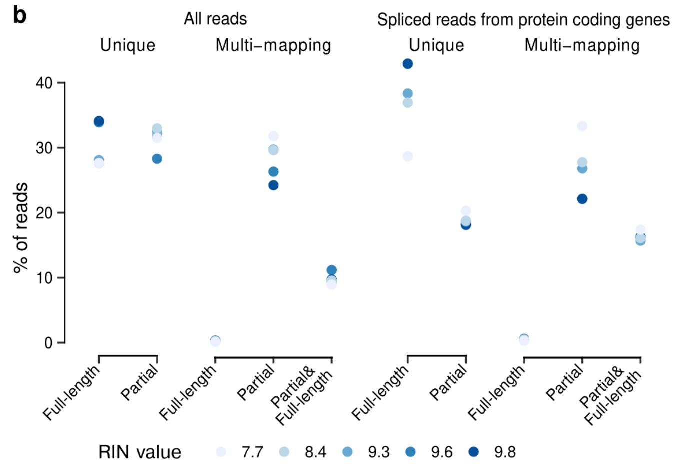

Supplementary Text Fig. 1. Percentage of full-length length reads in SG-NEx samples and publicly available data for different protocols
(a) Boxplots showing the percentage of reads being uniquely or multi-mapped to transcripts, and whether read is full-splice-junction matched to transcript or not (full-length vs partial) for all five protocols for spliced reads originating from protein coding genes in SG-NEx samples **(b)** Boxplots showing the percentage of reads being uniquely or multi-mapped to transcripts, and whether read is full-splice-junction matched to transcript or not (full-length vs partial) for all reads and spliced reads originating from protein coding genes for five different degraded direct RNA samples in Prawer et al study

The annotated start and end coordinates of the first and last exons are frequently different from what is observed in the data, and are therefore not suitable to estimate full length read counts [1,5–7]. To illustrate this, we have calculated the distance of direct RNA-Seq reads from the annotated 3' end of compatible transcripts. Direct RNA-Seq reads are sequenced from

the polyA tail, which ensures that all reads except sequencing and alignment artefacts cover the complete 3' end [8]. However, the average distance between the observed 3' end and polyAdenylation site from the direct RNA-Seq data and the annotated 3' end is 330 bp (Supplementary Text Fig. 2b), illustrating that start and end coordinates from annotations do not provide a reliable measure to identify full length reads. Therefore, here we used the definition of full splice match to define full length reads.

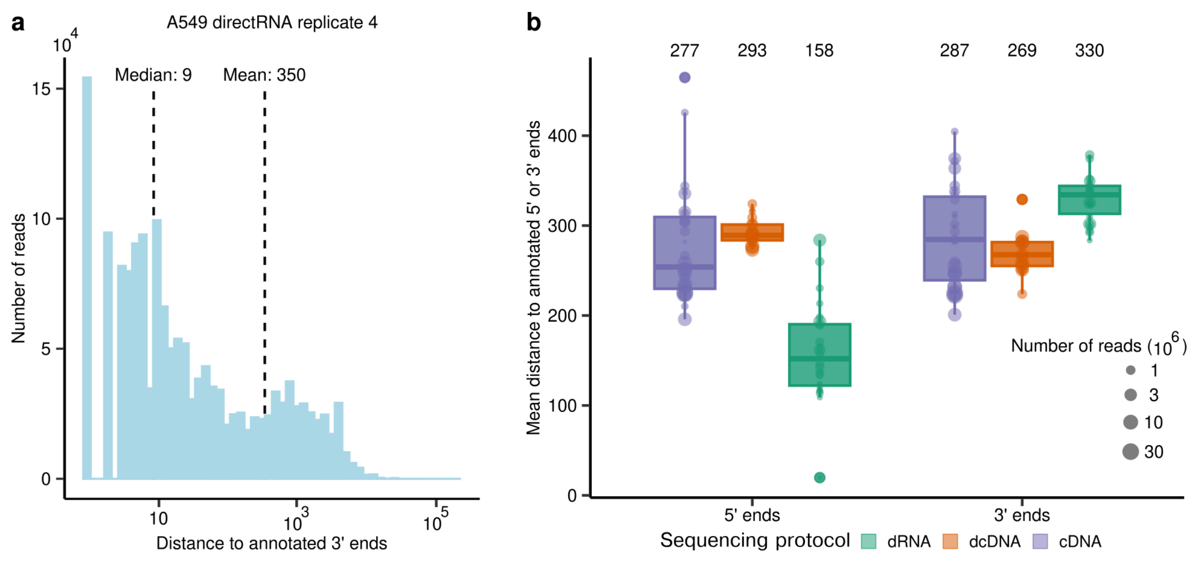

Supplementary Text Fig. 2 Average distance between observed start/ends and annotated start/ends for different RNA-seq protocols
(a) Histogram showing the distance to annoated 3' end for one replicate of A549 direct RNA sample, with mean and median indicated with dashed lines **(b)** Boxplots showing the median, upper and lower quartile, and 1.5 x interquartile ranges of the summarized mean distance to annotated 5' and 3' ends for each sample (direct RNA, n=55; direct cDNA, n=30; cDNA, n=27), point shape indicating number of reads generated for that sample

(2) Completely sequenced reads

Due to naturally occurring RNA degradation and the presence of novel genes and isoforms, some reads that do not match a full length transcripts (and which are therefore considered non-full length in the analysis) can still correspond to a completely sequenced reads. The PCR-cDNA protocol contains adapters at the 3' and 5' end, which can be used to identify reads that were completely sequenced.We estimated the fraction of completely sequenced reads by searching for the presence of 3' and 5' adapters in the PCR-cDNA protocol using Pychopper (https://github.com/epi2me-labs/pychopper). In total 91% of all reads have both adapters and correspond to completely sequenced reads (Supplementary Text Fig. 3). These results suggest that incomplete sequencing only contributes to a small fraction of non-full length reads.

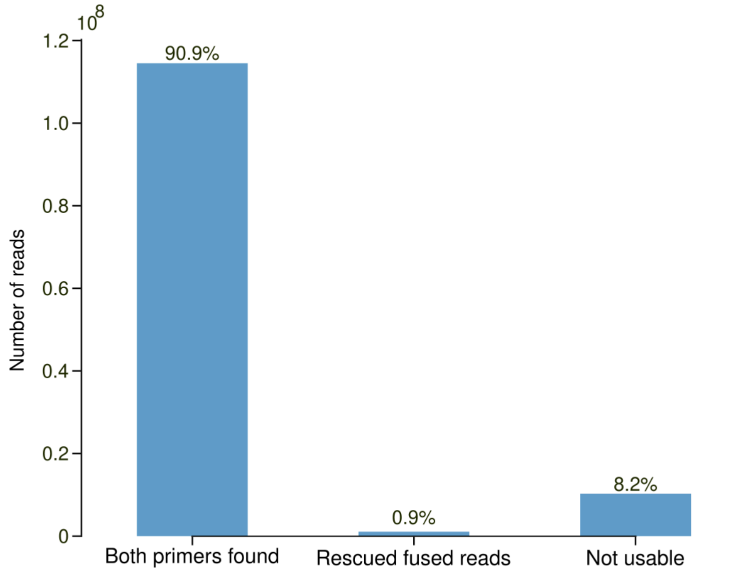

Supplementary Text Fig. 3 Percentage of reads contain both 5' and 3' primers for SGNex_Hct116_cDNA_replicate3_run5
Note:
Both primers found reads included reads containing strand-switching primer (SSP) and anchored oligo(dT) VN primer (VNP) in the correct orientation with default parameters in Pychopper
Rescued fused reads include reads containing multiple non-consecutive right configured primers' combinations[9]
Not usable reads include reads contain no right configured primers

(3) 5' and 3' end coverage

To further investigate non-full length reads, we analysed the fraction of reads that cover the 5' exon and the 3' exon of transcripts for the different long read protocols. As expected, the direct RNA-Seq data has the highest fraction of reads covering the 3' exon (median across all SG-NEx samples: 91%, Supplementary Text Fig. 4). However, even for the direct cDNA and the PCR-cDNA protocols, most reads cover the 3' exon (81% and 86% respectively) (Supplementary Text Fig. 4). In contrast, the 5' exon is covered less often (72%, 64%, 80%), possibly indicating the presence of RNA degradation or other protocol-specific limitations (Supplementary Text Fig. 4).

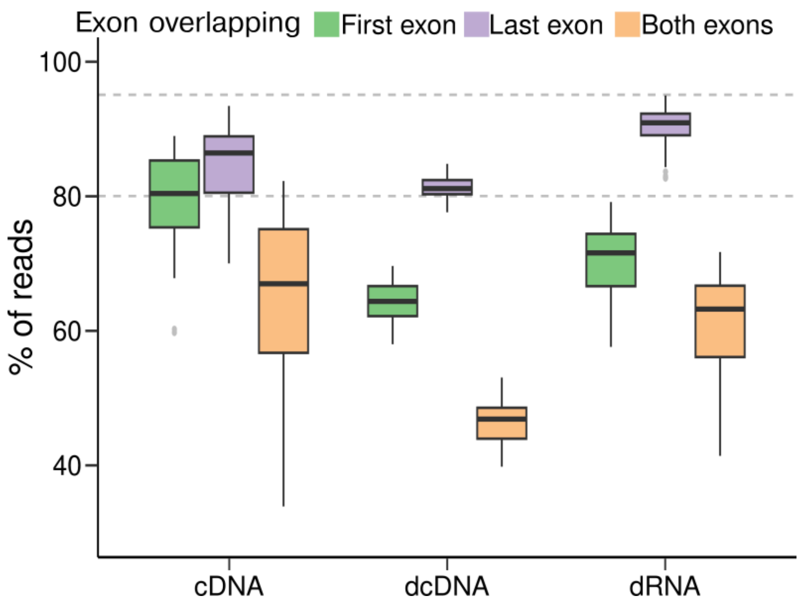

Supplementary Text Fig. 4 Percentage of reads covering starting and ending exons of transcripts for different RNA-seq protocols
Boxplots showing the median, upper and lower quartile, and 1.5 x interquartile ranges of the percentage of reads covering starting and ending exons for different RNA-seq protocol samples (direct RNA, n=55; direct cDNA, n=30; cDNA, n=27). Note here only spliced reads are considered for this analysis

2. Investigating incompatible reads observed in nanopore data

Long read RNA-Seq data has a higher number of reads that are incompatible with any transcript (number of transcripts = 0) when compared to short read RNA-Seq data. We have therefore investigated the cause for the larger fraction of reads that cannot be assigned to a transcript in long read RNA-Seq compared to short read RNA-Seq. Most of these reads use a combination of exons that is only observed by a single read (Median: 3.26%, IQR: 2.54%-6.8%, Range: 1.0%-16.5%, Supplementary Text Fig. 5a-c), which are supported by less than 5% of all reads which align to the respective gene (Mean: 1.0%, IQR: 0.5%-2.0%, Range: 0%-32%, Supplementary Text Fig. 5a-c), or which have Novel Discovery Rate (NDR) above the specified threshold in Bambu (Median: 2.9%, IQR: 2.2%-4%, Range: 1.2%-26.9%, Supplementary Text Fig. 5a-c). Furthermore, we have analysed highly accurate PacBio IsoSeq data for the same cell lines and found a similar fraction of reads that are not assigned to any transcript (Median: 15%, IQR:14%-16%), indicating this observations is not caused by the higher error rate in the nanopore sequencing data (Supplementary Text Fig. 5d).

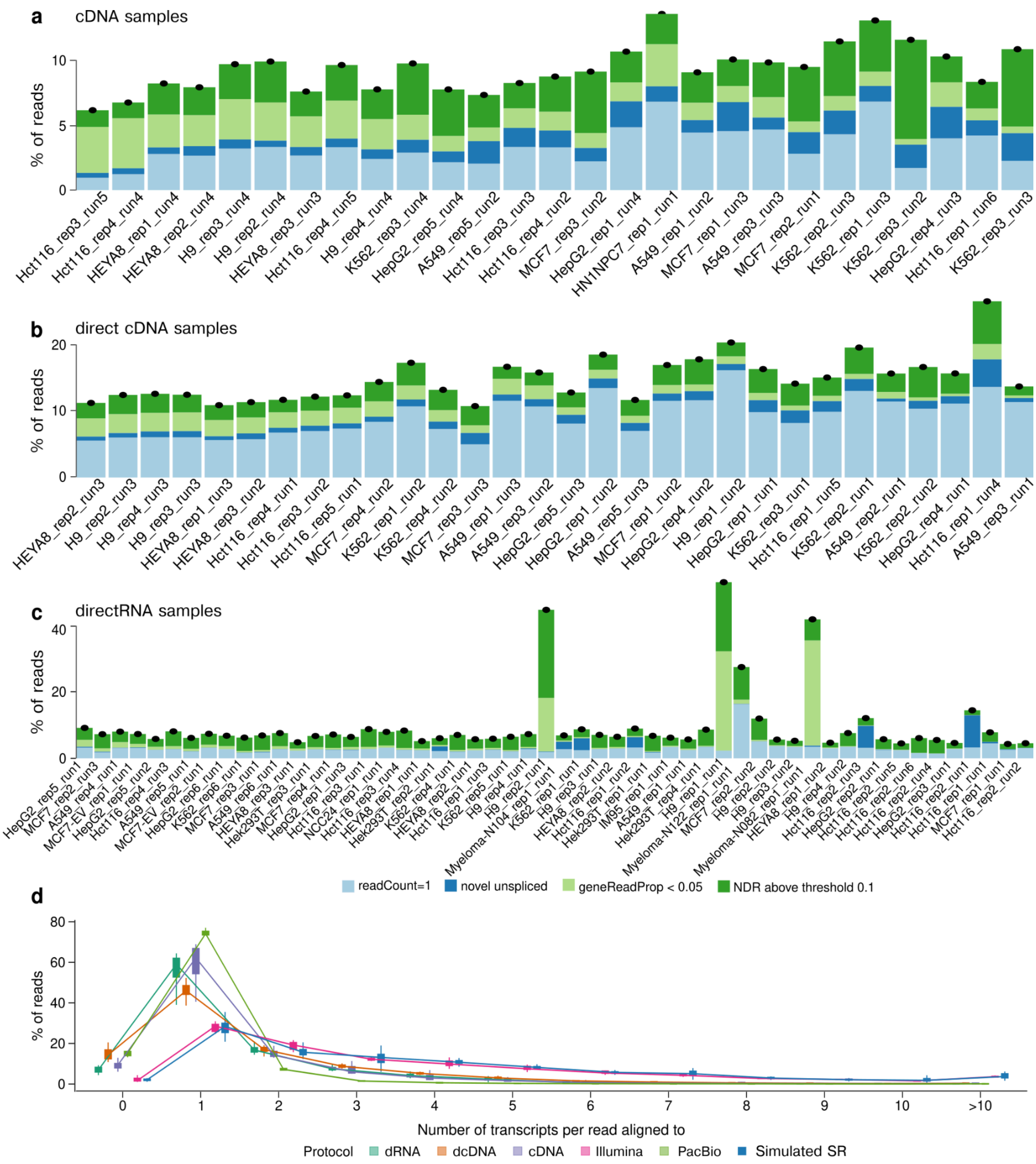

Supplementary Text Fig. 5 Investigation for reads incompatible to transcripts
(a-c) Percentage of incompatible reads for each sample classified by the filtering conditions for (a) cDNA, (b) direct cDNA, and (c) direct RNA samples **(d)** Boxplots showing the median, upper and lower quartile, and 1.5 x interquartile ranges of the number of transcripts per read assigned to for different RNA-seq protocols, and fragmentation simulated reads (direct RNA, n=55; direct cDNA, n=30; cDNA, n=27; PacBio, n=6; Illumina, n=21, simulated SR, n=67)

Next we tested if the fraction of unassigned reads is reduced when long read RNA-Seq data is fragmented to 150bp long reads (without error correction). Indeed, we find that the fragmentation reduces the fraction of unassigned reads to levels that are similar to short read RNA-Seq data, independently from the higher error rate (Fig. 4h-k, Supplementary Fig. 5o-p, Fragmented LR vs SR, 2.1% vs 1.8%).

Together, these results indicate that the difference in the fraction of unassigned reads between short and long read RNA-Seq data is not caused by a higher error rate, but due to read fragmentation which results in a higher number of compatible reads. Noteworthy, in case of the simulated read fragmentation, these reads will be wrongly assigned, suggesting that the low number of unassigned reads might be an artefact of short read RNA-Seq data.

3. Batch effect analysis

We have also performed sample correlation clustering with batch effects removed for protocol and cell line separately, using alternative methods in addition to Salmon. Here we present the results

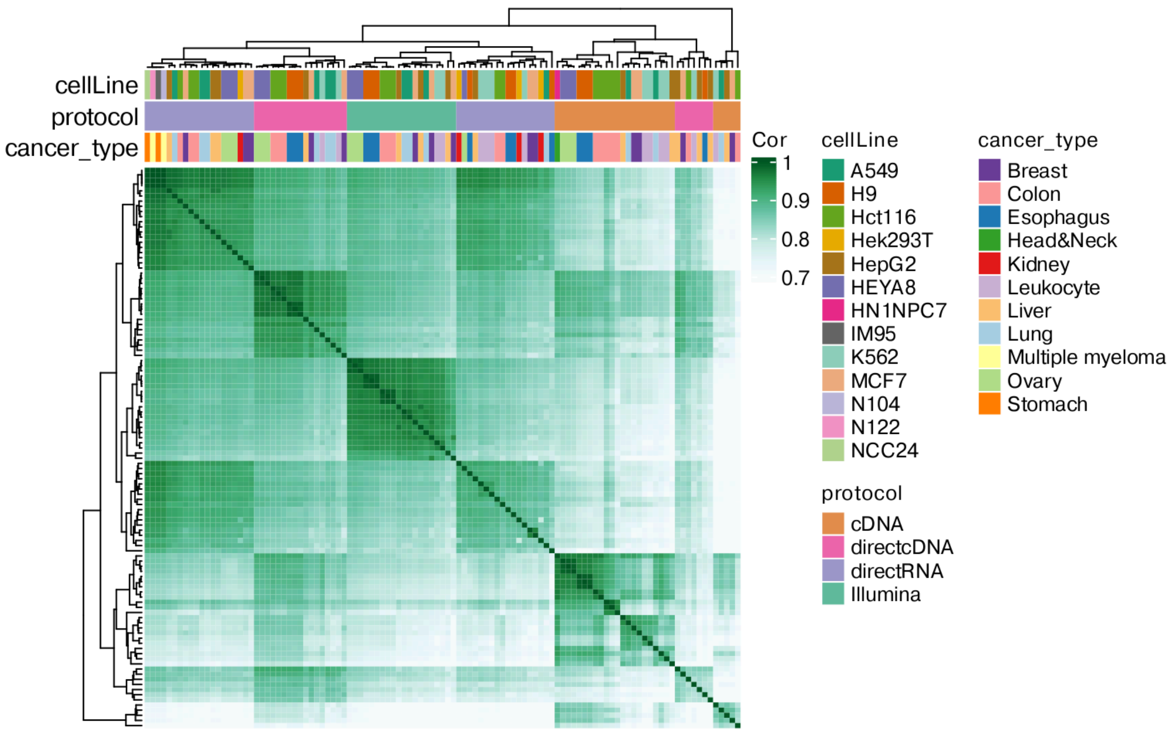

Supplementary Text Fig. 6 Heatmap showing the correlation of genes log2-transformed CPM estimates across the SG-NEx samples generated using PCR cDNA, direct cDNA, direct RNA and short read protocols when treating cell line as an batch effect

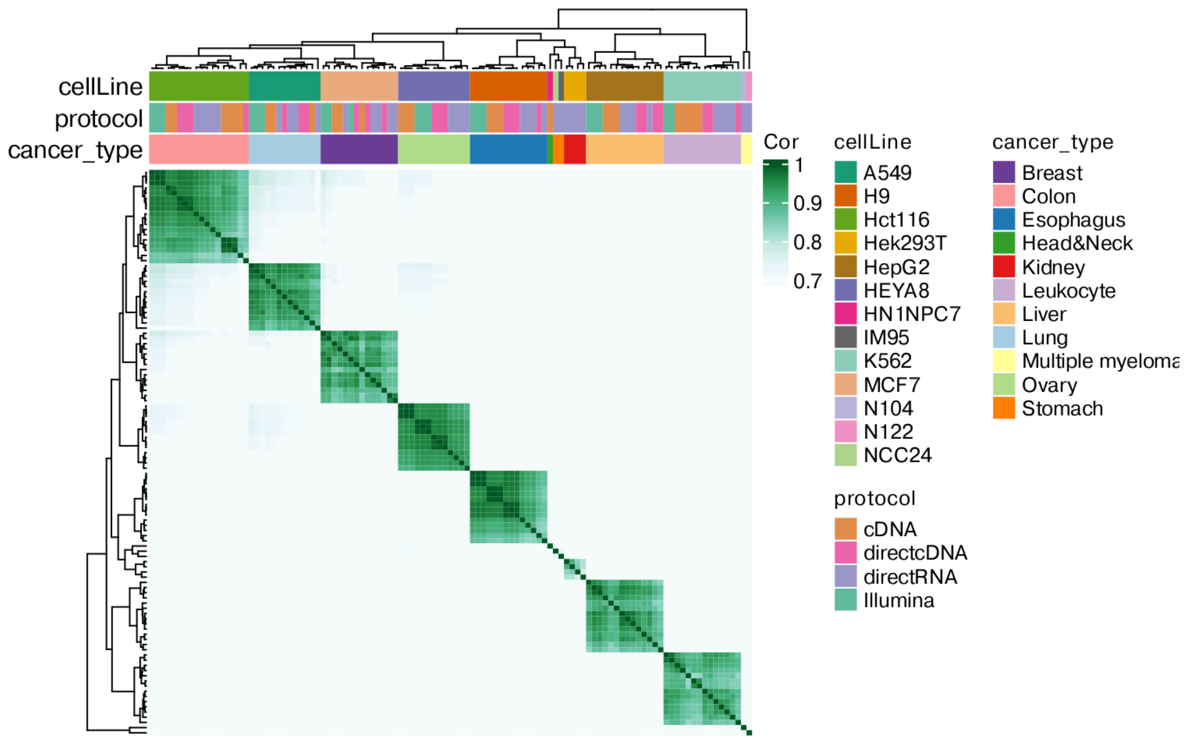

Supplementary Text Fig. 7 Heatmap showing the correlation of genes log2-transformed CPM estimates across the SG-NEx samples generated using PCR cDNA, direct cDNA, direct RNA and short read protocols when treating protocol as an batch effect

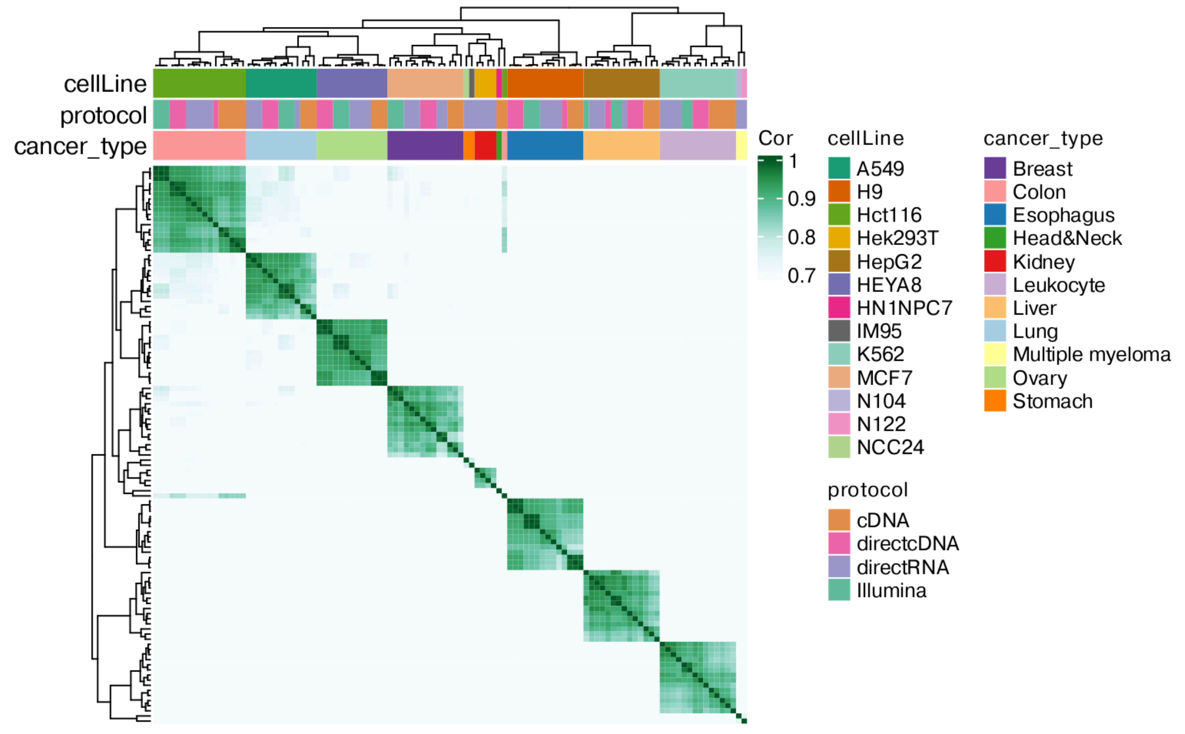

Supplementary Text Fig. 8 Heatmap showing the correlation of genes log2-transformed CPM estimates across the SG-NEx samples generated using PCR cDNA, direct cDNA, direct RNA and short read protocols using bambu for long read and RSEM for short read

4. Differential gene expression using long and short read RNA-seq data

To analyse the impact of technology on differential gene expression analysis when short and long read RNA-Seq data are combined, we have used DESeq2 [10] to identify differentially expressed genes across all pairs of the SG-NEx core cell lines for two different scenarios: (1) differential gene expression analysis using a single technology (2) combined-technology differential gene expression analysis (long + short read). To account for differences in read counts due to read fragmentation, we have converted CPM (long read) and TPM (short read) estimates to read count estimates, which were then used with DESeq2 [11,12](as recommended previously).

A comparison of the resulting log-fold changes shows a high level of correlation between short and long read RNA-Seq (Spearman correlation $\rho = 0.82$ to 0.93, Supplementary Text Fig. 9a), with the majority of differentially expressed genes being detected in both technologies (orange points). However, technology-specific differentially expressed genes were still detected (blue points).

Since the SG-NEx cell lines were sequenced with multiple replicates of different RNA-Seq protocols for each cell line, we could perform a combined analysis where the technology was specified as a batch effect in DESeq2. This combined analysis increased the correlation, leading to differential expression results that are highly similar compared to using only long read RNA-Seq data (0.94-0.97), indicating that the technology-specific effect can be moderated in a combined analysis (Supplementary Text Fig. 9b).

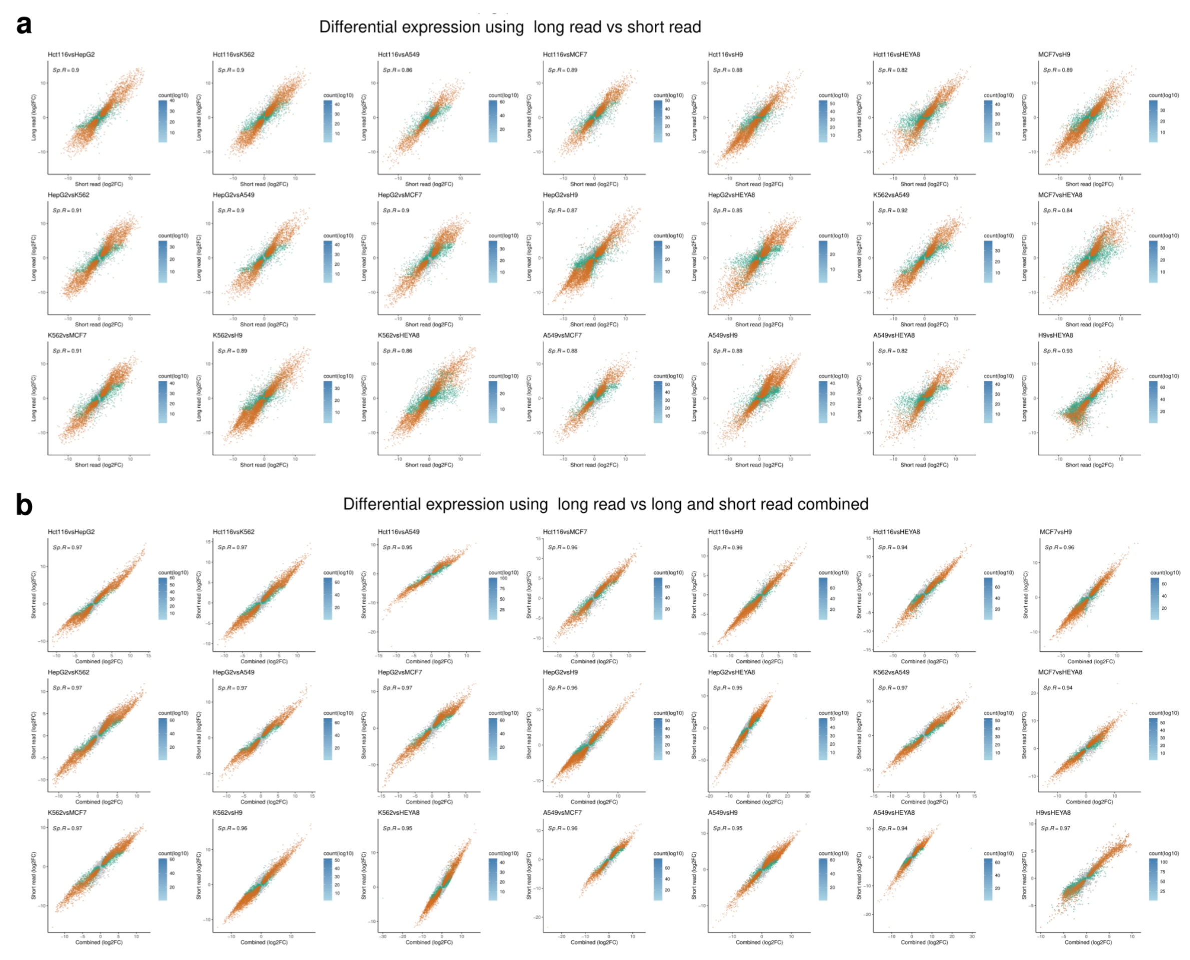

Supplementary Text Fig. 9 Differential expression results when using long and short read
(a) Scatter plots showing the log2 fold change when comparing gene expression estimates from two different cell lines using long read RNA-Seq against the log2 fold change using short read RNA-Seq. **(b)** Scatter plots showing the log2 fold change when comparing gene expression estimates from two different cell lines using long read RNA-Seq against the log2 fold change using combined long and short read RNA-Seq

5. Short read simulation

We simulated read fragmentation using the Illumina short read RNA-Seq data (150bp to 50bp read length, Supplementary Fig. 5p, Supplementary Text Fig. 10). While isoforms that were identified as major in both long and short read data were more robust against read fragmentation (ρ=0.88 (50 bp)), short-read specific major isoforms and minor isoforms were more strongly impacted (ρ=0.75 and ρ=0.59 respectively).

6. Paired-end short read simulation

The SG-NEx short read data consists of paired end, 150bp Illumina RNA-Seq. The main reason to simulate single end instead of paired end data is the variation in insert size, which is non-trivial to simulate as not all possible insert sizes are compatible with every transcript or long read (Supplementary Text Fig. 10a). We have now generated a simulated, paired end short read data set by sampling the insert size length from the empirical distribution, while limiting the insert size to be smaller than the observed long read length. The results

indeed show an even higher similarity with the Illumina paired end RNA-Seq data, confirming our observations from the single end read simulation (Supplementary Text Fig. 10b-c). Since the simulated single end short read data already shows a high similarity with the Illumina short read RNA-Seq data, we have not attempted to generate paired end simulations for all samples, even though we would expect that this might result in minor improvements.

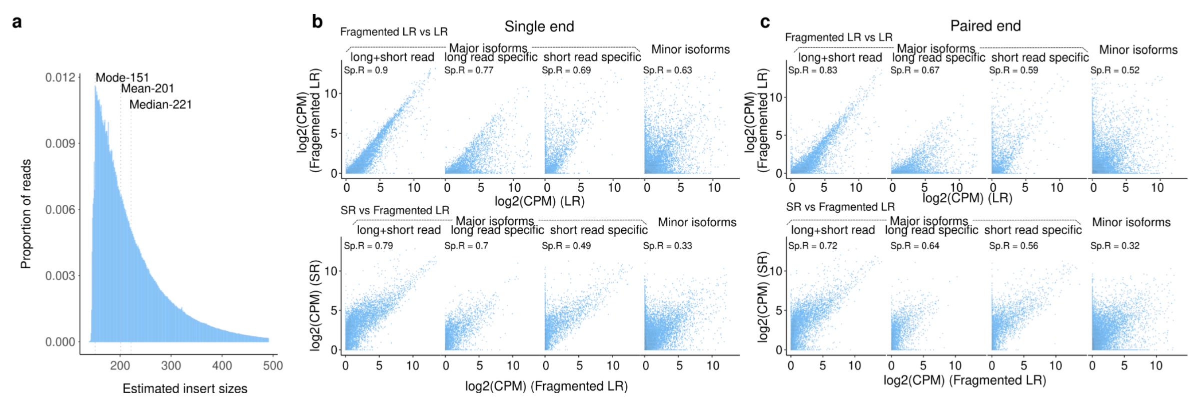

Supplementary Text Fig. 10 Paired end simulation results for one sample
(a) Estimated insert sizes based on “samtools stats” reported IS values on one short read sample **(b)** Scatterplots showing fragmentation simulated single-end short read against paired-end short read RNA-seq data or long read RNA-seq data **(c)** Scatterplots showing fragmentation simulated paired-end short read against paired-end short read RNA-seq data or long read RNA-seq data

7. Novel transcript candidates are confirmed using recent genome annotations

To investigate if these novel transcript candidates would be considered valid transcripts for genome annotations, we compared them with the most recent release of Gencode (GENCODE release 44, ENSEMBL release 110, released on July 2023) and RefSeq (Release 222, on Jan 16 2024). We found that 128 of novel transcript candidates were included in the most recent version of both human genome annotations and additional 495 were included in either of these two annotations, indicating that they are likely to be valid transcripts. Among these, 54 transcripts have a repeat content of 80% or higher in the previously unannotated exons sequence, and additional 7 transcripts have an overall repeat content of 80% or higher. Additionally, we further confirmed 11 novel transcripts, six non-repetitive (repeat overlapping percentage < 10%), five repetitive (repeat overlapping percentage >= 10%), with one highly repetitive (repeat overlapping percentage > 80%) (7 already included in RefSeq Release 222, and 4 not included in any of the recent annotations yet) using PCR and Sanger sequencing (Supplementary Text Fig. 11, Supplementary Table 9).

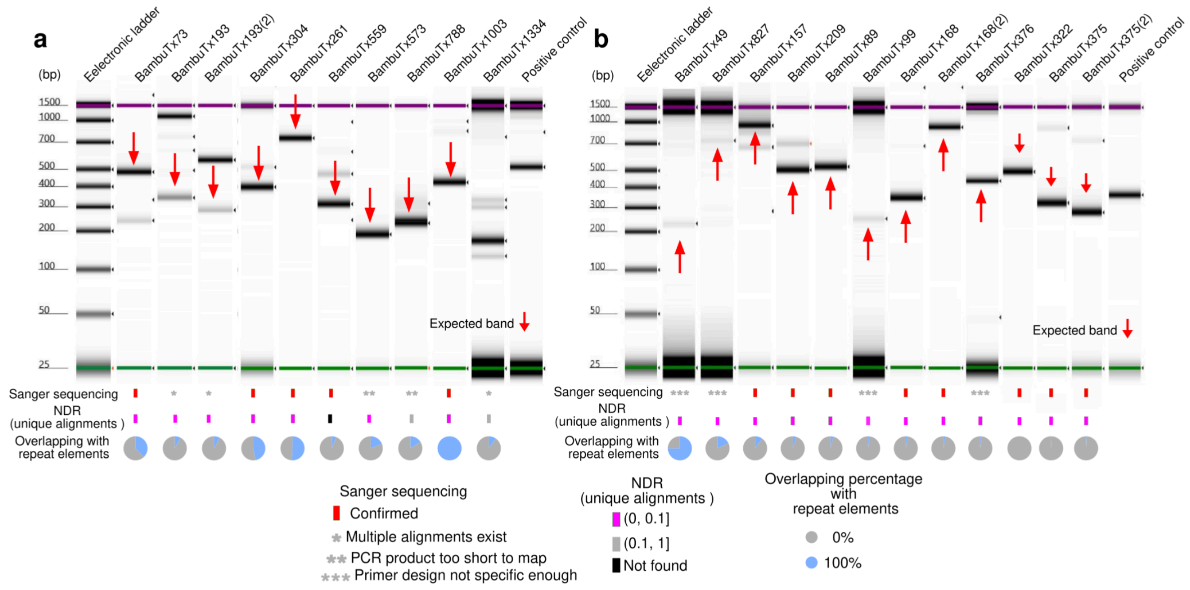

Supplementary Text Fig. 11 PCR bands observed for selected novel transcript candidates in MCF7 cell line

(a-b) PCR bands observed for selected novel transcript candidates in MCF7 cell line in two batches. Red arrows indicate the expected PCR band. The red bars indicate confirmed Sanger sequencing results. Single asterisk and double asterisks indicate inconclusive Sanger sequencing results due to multiple alignments existing and PCR product being too short to map respectively. Purple bars indicate novel transcripts identified with a NDR < 0.1 when using only reads with unique alignments. Grey bars indicate a NDR value between 0.1 and 1 when using only reads with unique alignments. Black bar indicate not identified when using only reads with unique alignments. The blue area in the pie charts represents the overlapping percentage with repeat elements. Due to the limited RNA left for validation, the validation experiment was done only once for each experiment. As an orthogonal check and a standard procedure, the sanger sequencing followed strengthened and confirmed some of the candidates.

8. Statistics for novel transcript candidates

To provide an overview of novel transcripts we compared their expression across the different samples, cell lines, and protocols. We observed that the vast majority of novel transcript candidates are found in multiple samples (Supplementary Text Fig. 12a) and across all three nanopore RNA-Seq protocols (Supplementary Text Fig. 12c), and that many novel transcripts can be detected in multiple cell lines (Supplementary Text Fig. 12b). The expression level is lower compared to annotated genes, but comparable across cell lines and protocols (Supplementary Text Fig. 12d)

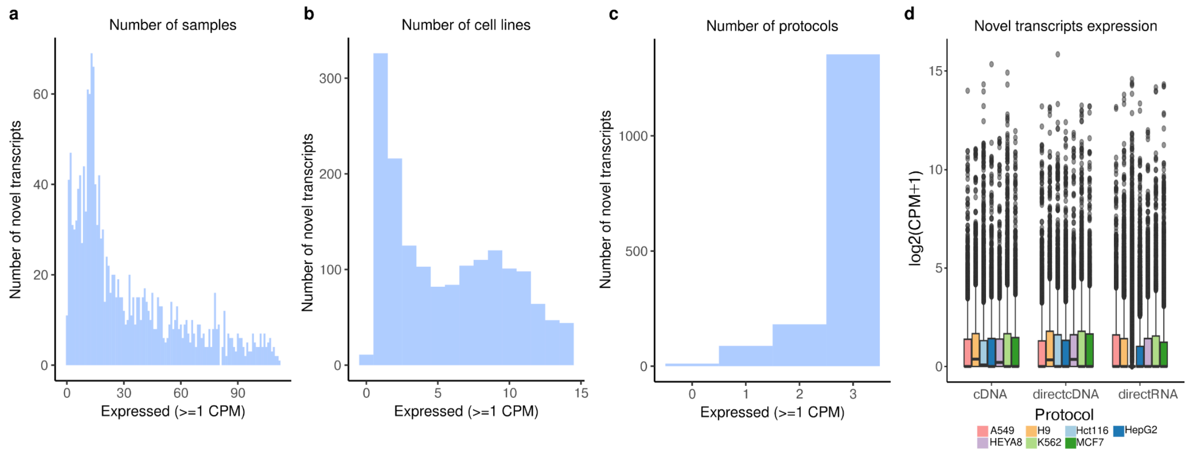

Supplementary Text Fig. 12 Novel transcripts detected in multiple samples, cell lines and protocols
(a-c) Histograms showing the frequency of novel transcripts that are detected in varying number of (a) samples, (b) cell lines and (c)protocols **(d)** Boxplots showing the median, upper and lower quartile, and 1.5 x interquartile ranges of the log2 transformed CPM of novel transcripts in cDNA (n=4905 for A549, H9, HepG2, HEYA8, MCF7, 8175 for K562, and 9810 for Hct116), direct cDNA (n=4905 for HEYA8 and MCF7, 6540 for H9, and 8175 for A549, Hct116, HepG2, K562), direct RNA (n=6540 for A549, K562, 8175 for HEYA8, 9810 for HepG2, 11445 for H9 and MCF7, and 21255 for Hct116) samples for each cell line.

9. PacBio comparison

When analysing gene and transcript quantification across different RNA-seq protocols, the PacBio IsoSeq data shows lower correlation with short read RNA-Seq (median spearman correlation ρ = 0.79 for spike-ins) and with Nanopore long read RNA-Seq (median ρ =0.61 for spike-ins). This observation is likely due to an under-representation of shorter transcripts (Supplementary Text Fig. 13).

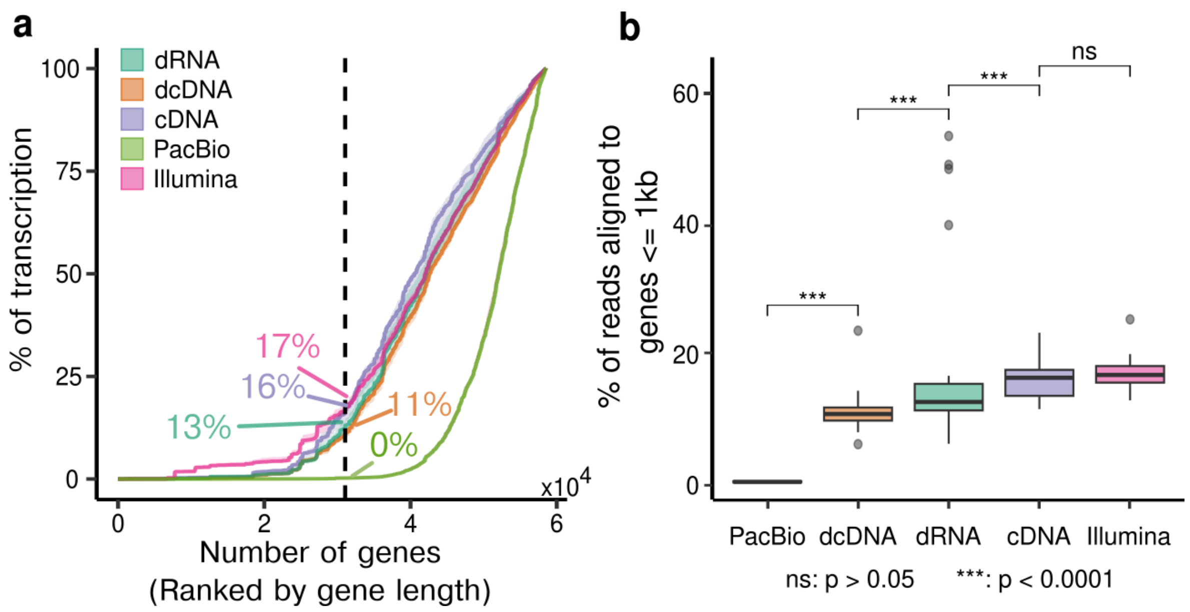

Supplementary Text Fig. 13 Transcription by gene length for different sequencing protocols
(a) Cumulative distribution plots for percentage of transcription when ranking genes by gene length for direct RNA-Seq (dRNA), direct cDNA (dcDNA), PCR cDNA (cDNA), PacBio long read RNA-Seq and Illumina short

read RNA-Seq **(b)** Boxplots showing the median, upper and lower quartile, and 1.5 x interquartile ranges of fraction of transcription the top genes with gene length less than 1kb for direct RNA-Seq (dRNA, n=55), direct cDNA (dcDNA, n=30), PCR cDNA (cDNA, n=27), PacBio long read RNA-Seq (n=6) and Illumina short read RNA-Seq (n=21), with two-sided t-test p-values indicated as ns for non significant, and *** for p-value <=0.0001

10. Replicability using the same platform and protocol

We compared gene and transcript expression estimates across biological replicates from each protocol to evaluate the replicability within each technology. Here, short read RNA-Seq showed higher correlation across replicates compared to the nanopore long read RNA-Seq protocols both for gene and transcript expression (Supplementary Text Fig. 14). When only protein coding genes are considered, replicability improves for all RNA-Seq protocols Supplementary Text Fig. 14a). For transcript expression, a higher correlation across biological replicates was observed for all protocols when only major isoforms were considered (Supplementary Text Fig. 14b). These results indicate that replicability within the same technology is higher for short read RNA-Seq compared to nanopore long read RNA-Seq, possibly reflecting a higher variation in throughput, among others.

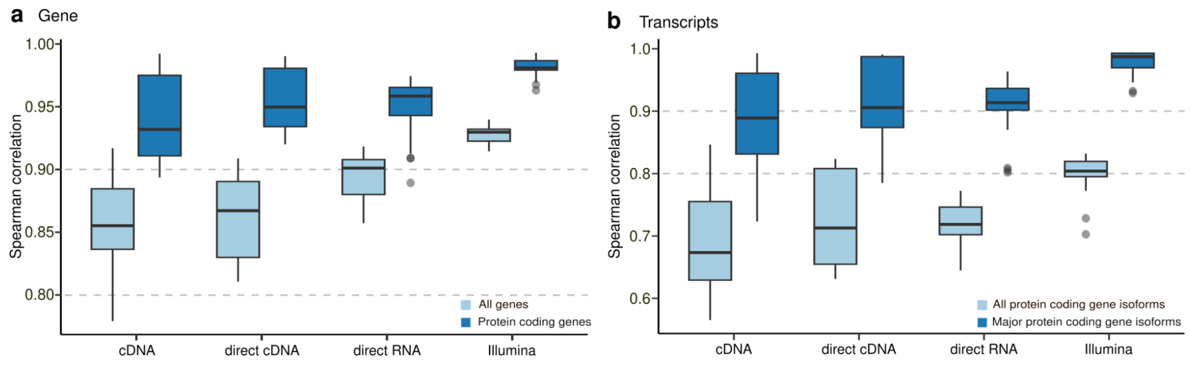

Supplementary Text Fig. 14 Reproducibility between biological replicates
(a-b) Boxplots showing the median, upper and lower quartile, and 1.5 x interquartile ranges of the spearman correlation between biological replicates (n=33,29,46,21 for cDNA, direct cDNA, direct RNA and Illumina, respectively) for a) gene expression and b) transcript expression. For transcript expression, only transcripts from proteining coding genes are included.

11. Pseudogenes

We observed that long read RNA-Seq data has a higher fraction of reads aligned to pseudogenes. Across all samples, we observe that 3.5% of primary read alignments are assigned to pseudogenes (Supplementary Text Fig. 15). When we discard all reads with multiple alignments, this fraction is reduced to 0.2% (Supplementary Text Fig. 15), reflecting that pseudogenes expression estimates largely originate from multi-mapping reads (Supplementary Text Fig. 15). To test if the sequencing or alignment error could explain the observed read count for pseudogenes, we compared the results to PacBio IsoSeq data, which has a lower error rate. The PacBio data shows a lower fraction of reads being aligned to pseudogenes compared to the Nanopore RNA-Seq data (0.1%, Supplementary Text Fig.

15), suggesting that alignment errors might partially explain the observed read count for pseudogenes.

In contrast to pseudogenes, which are highly similar to the original gene copy, transcripts which overlap repetitive elements such as LINE, SINE, and LTR transposons often have some unique sequence part, and we do not observe that multi-mapped reads have an influence on these results. We would also like to note that pseudogenes are included in reference annotations and are therefore not considered novel transcripts, and they are also not included in the list of repetitive elements which is based on RepeatMasker.

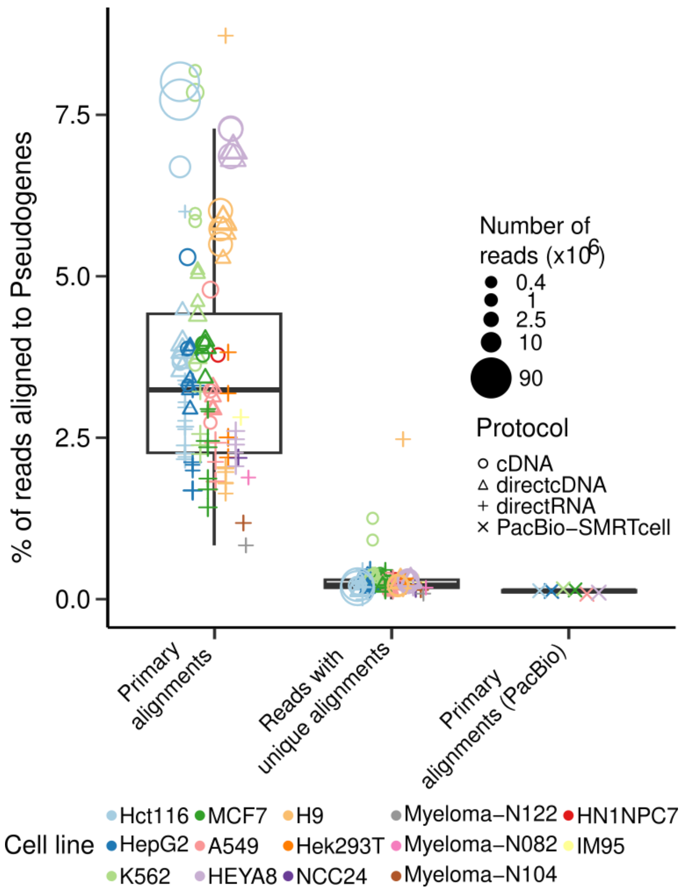

Supplementary Text Fig. 15 Percentage of reads aligned to Pseudogenes across samples when primary alignments, reads with unique alignments, or PacBio reads are used
Boxplots showing the median, upper and lower quartile, and 1.5 x interquartile ranges of the percentage of reads aligned to pseudogenes across SG-NEx Nanopore samples (n=112) when using primary alignments, reads with unique alignments, or across PacBio samples (n=6) when PacBio reads are used

12. Variation in sequencing depth for the nanopore long-read RNA-seq data

Sequencing depth shows higher variation for the nanopore long read RNA-Seq data compared to the short read RNA-Seq data. The five main factors that cause this variation are (1) that the different protocols (direct RNA-Seq, direct cDNA-Seq, PCR-cDNA-Seq)

generate different read numbers, (2) that the sequencing devices (MinION/GridION vs PromethION) generate different throughput, (3) that multiplexing was used for some samples, whereas other samples were sequenced without multiplexing, (4) that some samples were sequenced multiple times and then combined (technical replicates), whereas other samples were sequenced only once, and (5) that improvements in sequencing chemistry have led to higher throughput in the more recent data sets (e.g PCS109 kit vs PCS110 kit).

13. Novel transcripts are enriched in retrotransposons

Compared to annotated transcripts, we observed a significant enrichment of repetitive elements in exons from novel transcripts, both when all repeats are considered and when only LTR, LINE, and SINE retrotransposons are considered (Supplementary Fig. 7d, Supplementary Text Fig. 17, see methods). The enrichment of these highly repetitive elements was observed for both novel transcripts from annotated genes and for novel gene candidates, and was confirmed when transcript discovery was performed after multi-mapping reads were excluded (Supplementary Fig. 7c and 8f-i).

14. Transcripts overlapping with repeat elements with different thresholds

Here we checked the fraction of LTR, LINE, and SINE elements when different thresholds are used. We first compared the distribution of overlapping percentages for all transcripts and transcripts with at least 80% overlapping with repeat elements. We found that the results were similar with LTR being the largest group (Supplementary Text Fig. 16). We also presented an overview that shows the overlap of all different repetitive elements (from RepeatMasker, Supplementary Text Fig. 17) which are used in our analyses for all novel transcripts, and for novel transcripts with a minimum of 80% overlap with repeats.

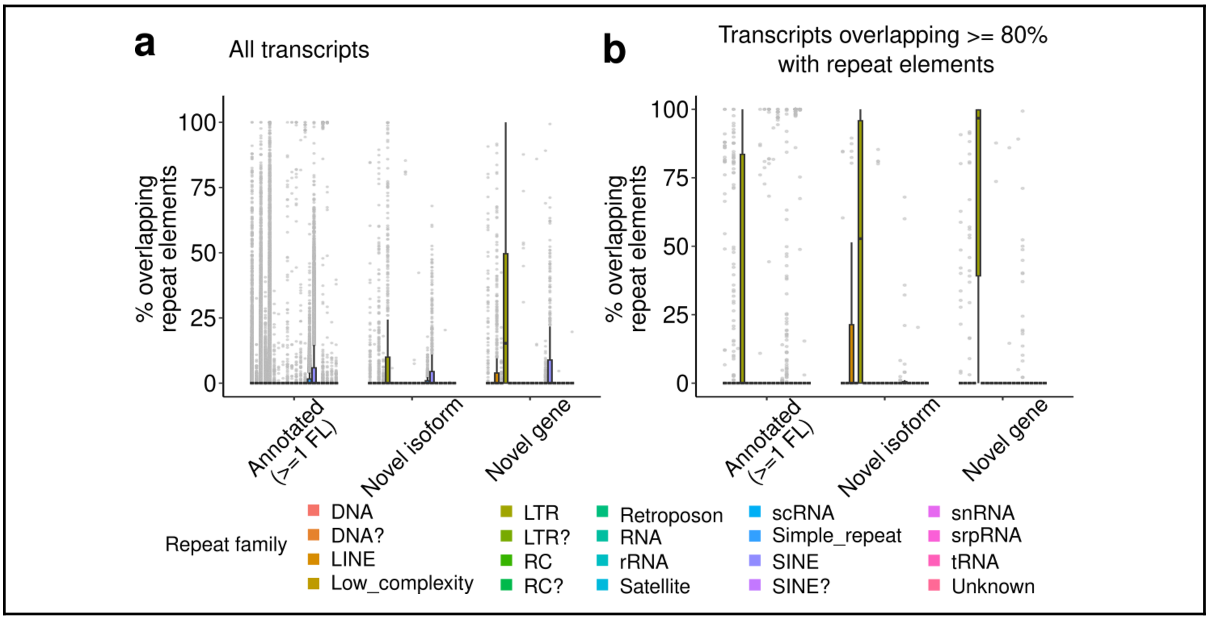

Supplementary Text Fig. 16 Distribution of overlapping percentages for all transcripts and transcripts with at least 80% overlapping with repeat elements
(a-b) Boxplots showing the median, upper and lower quartile, and 1.5 x interquartile ranges of overlapping percentages for (a) all transcripts and (b) transcripts with at least 80% overlapping with repeat elements for annotated transcripts with at least 1 full-splice-match read support across samples (Annotated >=1 FL, n=74455), all novel isoforms (n=908), and all novel gene isoforms (n=617).

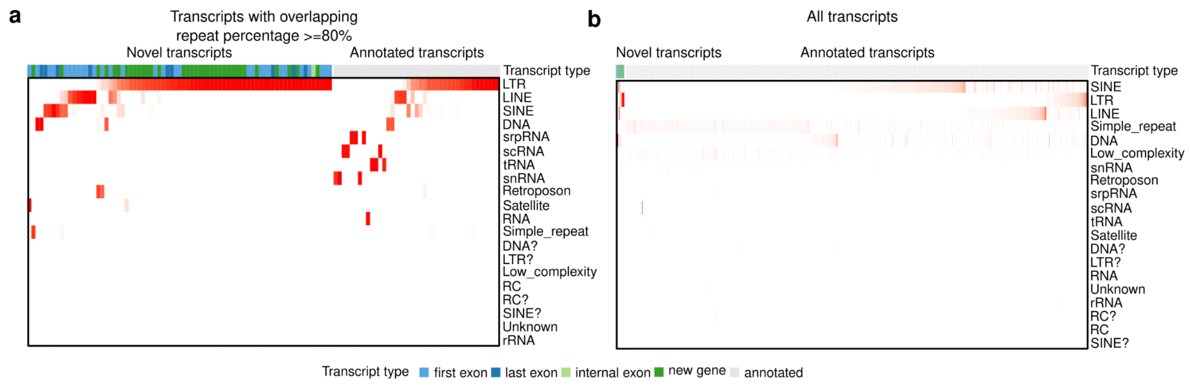

Supplementary Text Fig. 17 Overview of the repeat family in expressed transcripts
(a-b) Overview with repeat family in (a) transcripts at least 80% overlap with repeat elements expressed with >= 20 CPM in any samples and (b) all transcripts expressed with >= 20 CPM in any samples.

15. Analysis of multi-mapped reads and their impact on transcript discovery

We performed random downsampling from aligned reads of one sample to obtain a similar number of reads after using only unique alignments (using “samtools -s ”). We then compared the transcript discovery results when using 1) primary read alignments, 2) unique alignments, and 3) randomly downsampled alignments (Supplementary Text Fig. 18). We found that when a similar fraction of reads is sampled regardless of the mapping ambiguity, a lower reduction of novel transcripts was observed (unique vs downsampling: 6326 vs 6984 when a NDR threshold of 1 is used). However, as multi-aligned reads will be included, this downsampling strategy is unable to control for possible artefacts from such reads.

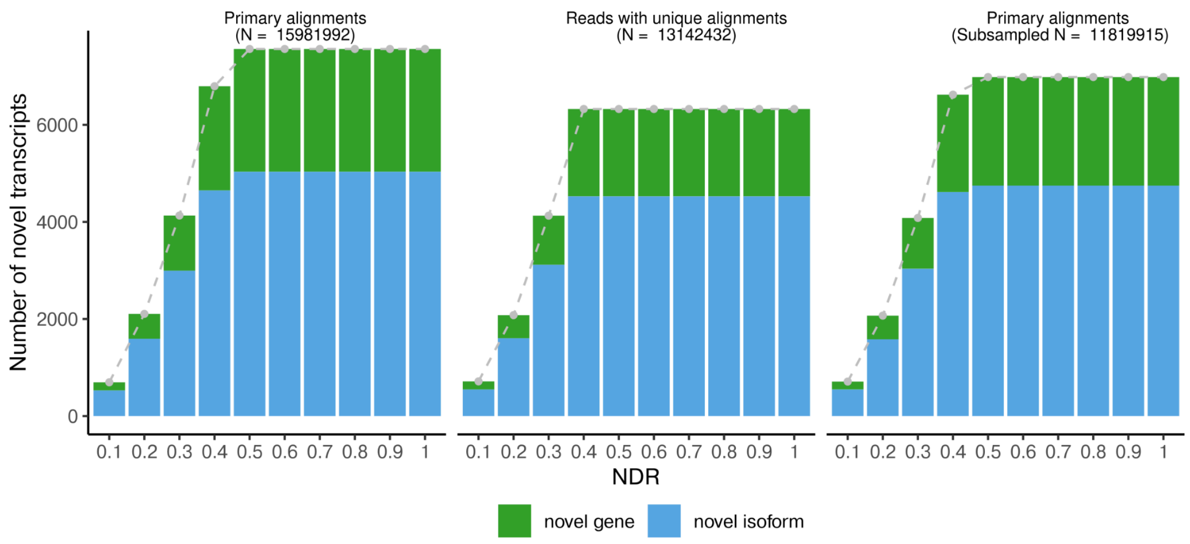

Supplementary Text Fig. 18 Impact of multiple-mapping reads on transcript discovery
Barplots presenting the number of novel transcripts identified when primary alignments of all reads are used vs reads with unique alignments vs primary alignments of a subset of all reads are used with varying transcript discovery threshold ranging from 0.1 to 1, at an increasing step of 0.1 Blue bars represent the number of novel isoforms of annotated genes discovered, green represents the number of novel isoforms of novel genes discovered. The grey points represent the total number of novel transcripts.

16. Analysis for lowly expressed spike-in genes

We compared gene expression quantification among different RNA-Seq methods with spike-in RNAs specifically for those that are lowly expressed (CPM<2.5, Supplementary Text Fig. 19). While the data show that gene expression estimates have higher variation for lowly expressed genes, the overall results remain similar, with both short read and long read achieving similar accuracy on spike-in RNAs for gene expression estimates (Supplementary Text Fig. 19).

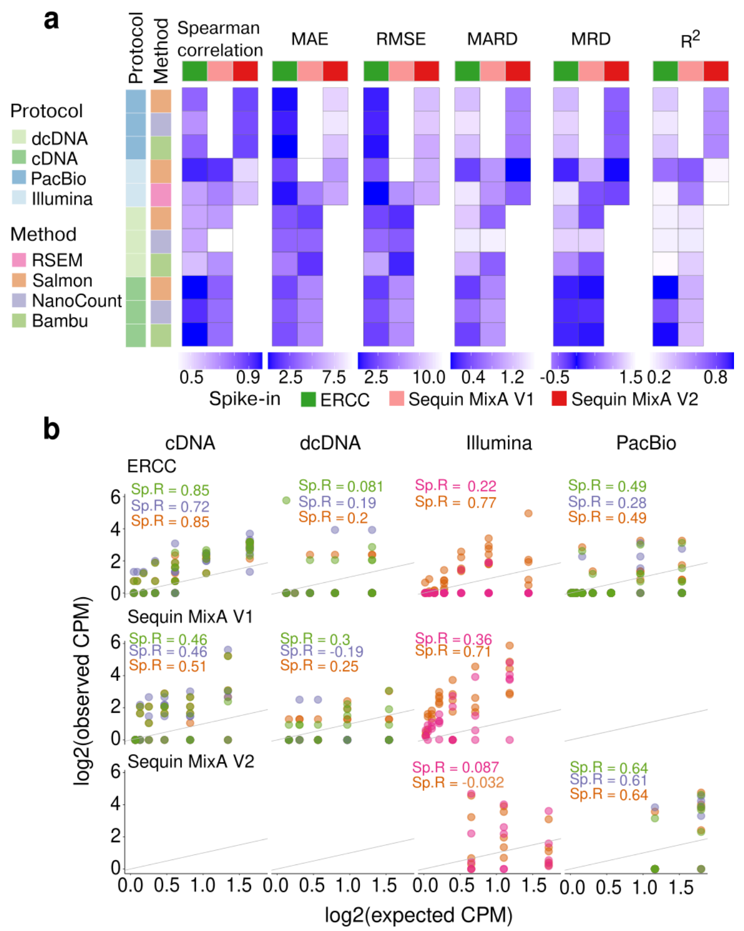

Supplementary Text Fig. 19 Analysis of spike-in RNAs with expected CPM < 2.5.
(a-b) Gene expression correlation metrics for spike-in RNAs with expected CPM < 2.5. The correlation coefficients still suggest long read and short read RNA-Seq are comparable in for estimating gene expression for spike-in RNAs. Note that expected CPM are calculated based on spike-in percentage and sequencing depth, which shows variation across protocols, hence, the expected CPM of the same spike-in RNA may be different in different protocols. For this analysis, only ERCC and Sequin RNAs have transcripts below the specified threshold.

17. Comparison of transcript expression using ENCODE short read RNA-seq data

To demonstrate that our results are not an artefact of the specific analysis methodology used for data processing in this manuscript, we downloaded the already processed short read RNA-Seq data from ENCODE for the A549, HepG2, K562, MCF7, and Hct116 cell lines [13] (Supplementary Text Table 1). For these datasets, transcript expression was quantified using two methods: Kallisto [14] and RSEM [15]. We then compared these estimates against the original long read RNA-Seq data and the simulated short read data obtained from fragmentation of long read RNA-Seq data (fragmented long read RNA-Seq data). We find that the results are highly similar to those observed with the SG-NEx short read RNA-Seq data. Both short read RNA-Seq datasets were more correlated with the fragmented long read RNA-Seq data than the original long read RNA-Seq data (see Supplementary Text Fig. 20). Across all datasets, we observed the strongest increase in correlation for short-read specific major isoforms (Two-sided Mann-Whitney U test $p < 0.0001$). This provides

additional support for our hypothesis that read fragmentation partially explains the observed differences in transcript expression estimates between short read and long read RNA-Seq data.

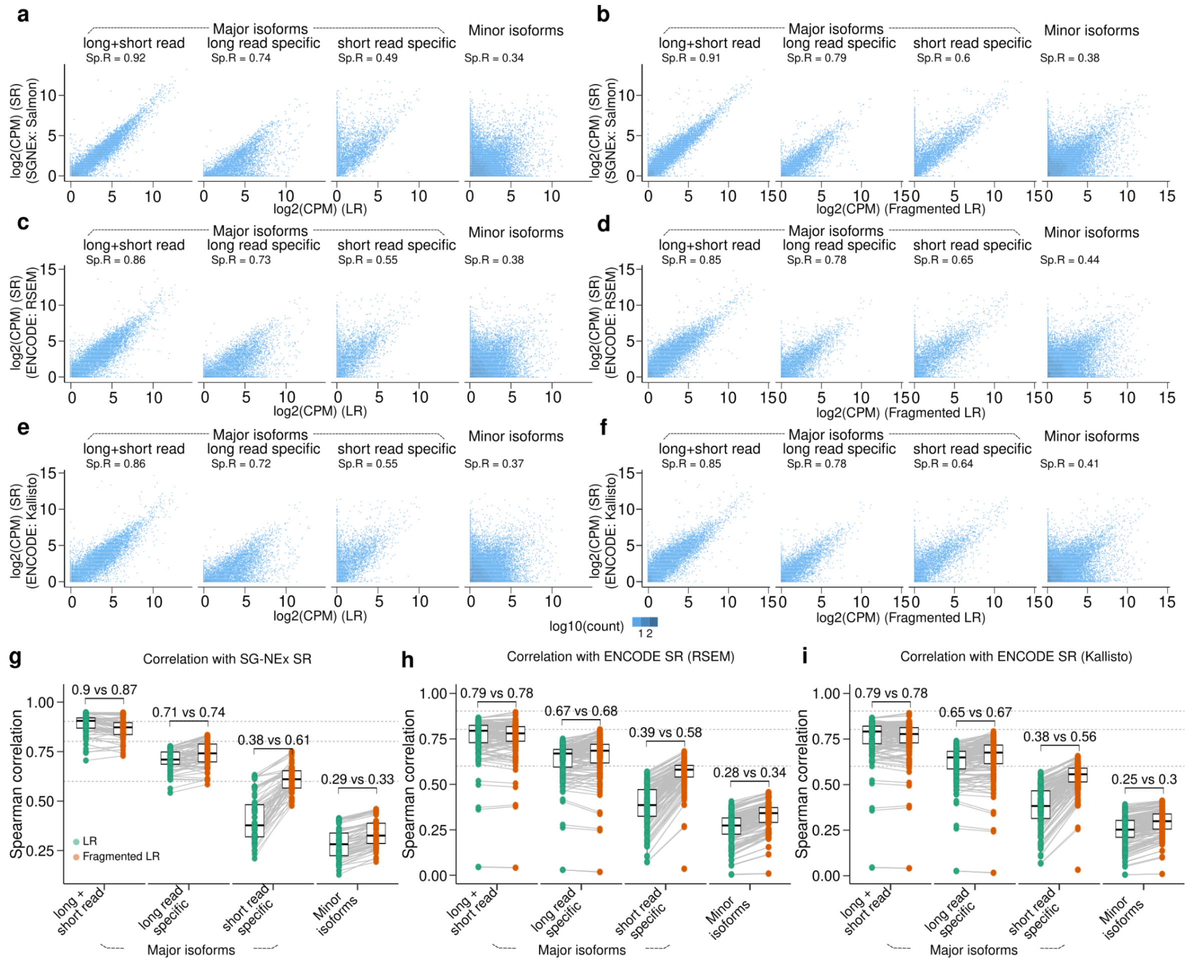

Supplementary Text Fig. 20 ENCODE short-read RNA-Seq data and SG-NEx short-read RNA-Seq data showed similar correlations with both long-read original and fragmented data for transcript expression **(a-b)** Scatterplots of log2-transformed CPM for protein-coding gene isoforms obtained from (a) long read direct cDNA RNA-Seq (using Salmon) and (b) fragmentation simulated short read from direct cDNA RNA-Seq (using Salmon with bias correction) against that obtained from short read RNA-Seq (using Salmon with bias correction) in the A549 cell line are shown for major isoforms identified in both long and short read (Major isoform: long + short read), isoforms major only in long read (Major isoform: long read specific), isoforms major only in short read (Major isoform: short read specific) and isoforms that are not major isoforms (Minor isoforms). **(c-d)** Scatterplots of log2-transformed CPM for protein-coding gene isoforms obtained from (c) long read direct cDNA RNA-Seq (using Salmon), or (d) fragmentation simulated short read from direct cDNA RNA-Seq (using Salmon with bias correction) against that obtained from ENCODE short read RNA-Seq (using RSEM) in the A549 cell line are shown for are shown for major isoforms identified in both long and short read (Major isoform: long + short read), isoforms major only in long read (Major isoform: long read specific), isoforms major only in short read (Major isoform: short read specific) and isoforms that are not major isoforms (Minor isoforms). **(e-f)** Scatterplots of log2-transformed CPM for protein-coding gene isoforms obtained from (e) long read direct cDNA RNA-Seq (using Salmon), or (f) fragmentation simulated short read from direct cDNA RNA-Seq (using Salmon with bias correction) against that obtained from ENCODE short read RNA-Seq (using Kallisto) in the A549 cell line are shown for are shown for major isoforms identified in both long and short read (Major isoform: long + short read), isoforms major only in long read (Major isoform: long read specific), isoforms major only in short read (Major isoform: short read specific) and isoforms that are not major isoforms (Minor isoforms).**(g)** Boxplots showing the median, upper and lower quartiles, and 1.5 x interquartile range of the mean absolute error (MAE) between short read (SR) and long read (LR), as well as in-silico simulated short read RNA-Seq data (simulated SR) for major isoforms identified in both long and short read (Major isoform: long + short read), isoforms major only in long read (Major isoform: long read specific),

isoforms major only in short read (Major isoform: short read specific) and isoforms that are not major isoforms (Minor isoforms). Light grey lines connect the same direct cDNA RNA-Seq sample (n=67) **(h-i)** Boxplots showing the median, upper and lower quartiles, and 1.5 x interquartile range of the mean absolute error (MAE) between (h) ENCODE short read (RSEM) or (i) ENCODE short read (Kallisto) and long read (LR) or in-silico simulated short read RNA-Seq data (simulated SR) for major isoforms identified in both long and short read (Major isoform: long + short read), isoforms major only in long read (Major isoform: long read specific), isoforms major only in short read (Major isoform: short read specific) and isoforms that are not major isoforms (Minor isoforms). Light grey lines connect the samples from ENCODE to direct cDNA RNA-Seq data in the same cell line (n=154).

Cell line	Replicate	File name	Sample link	transcript_tsv_kallisto	transcript_tsv_rsem	gene_tsv_rsem
A549	Rep1	ENCFF000EJJ	https://www.encodeproject.org/experiments/ENCSR000CON/	https://www.encodeproject.org/files/ENCFF213OJN/@@download/ENCFF213OJN.tsv	https://www.encodeproject.org/files/ENCFF081TVM/@@download/ENCFF081TVM.tsv	https://www.encodeproject.org/files/ENCFF855AKQ/@@download/ENCFF855AKQ.tsv
		ENCFF000EJV				
	Rep2	ENCFF000EJW		https://www.encodeproject.org/files/ENCFF226PTY/@@download/ENCFF226PTY.tsv	https://www.encodeproject.org/files/ENCFF329YJQ/@@download/ENCFF329YJQ.tsv	https://www.encodeproject.org/files/ENCFF244DNJ/@@download/ENCFF244DNJ.tsv
		ENCFF000EKB				
Hct116	Rep1	ENCFF000DKT*(incomplete filenames)	https://www.encodeproject.org/experiments/ENCSR000CWM/	https://www.encodeproject.org/files/ENCFF654QMA/@@download/ENCFF654QMA.tsv	https://www.encodeproject.org/files/ENCFF003PGO/@@download/ENCFF003PGO.tsv	https://www.encodeproject.org/files/ENCFF906KQM/@@download/ENCFF906KQM.tsv
		ENCFF000DKU*(incomplete filenames)				
	Rep2	ENCFF000DKZ*(incomplete filenames)		https://www.encodeproject.org/files/ENCFF964VPA/@@download/ENCFF964VPA.tsv	https://www.encodeproject.org/files/ENCFF651GQO/@@download/ENCFF651GQO.tsv	https://www.encodeproject.org/files/ENCFF092ZUO/@@download/ENCFF092ZUO.tsv
		ENCFF000DLA*(incomplete filenames)				
HepG2	Rep1	ENCFF000FVT	https://www.encodeproject.org/experiments/ENCSR000CPE/	https://www.encodeproject.org/files/ENCFF642NRP/@@download/ENCFF642NRP.tsv	https://www.encodeproject.org/files/ENCFF416RHJ/@@download/ENCFF416RHJ.tsv	https://www.encodeproject.org/files/ENCFF168QKW/@@download/ENCFF168QKW.tsv
		ENCFF000FVU				
	Rep2	ENCFF000FVI		https://www.encodeproject.org/files/ENCFF977QAI/@@download/ENCFF977QAI.tsv	https://www.encodeproject.org/files/ENCFF938LTW/@@download/ENCFF938LTW.tsv	https://www.encodeproject.org/files/ENCFF831QQF/@@download/ENCFF831QQF.tsv
		ENCFF000FVV				
K562	Rep1	ENCFF001RDZ	https://www.encodeproject.org/experiments/ENCSR000AEM/	https://www.encodeproject.org/files/ENCFF741NSE/@@download/ENCFF741NSE.tsv	https://www.encodeproject.org/files/ENCFF337XTV/@@download/ENCFF337XTV.tsv	https://www.encodeproject.org/files/ENCFF222UVT/@@download/ENCFF222UVT.tsv
		ENCFF001RED				
	Rep2	ENCFF001REF		https://www.encodeproject.org/files/ENCFF752DDP/@@downlo	https://www.encodeproject.org/files/ENCFF173RJM/@@downloa	https://www.encodeproject.org/files/ENCFF742CVV/@@downloa

		ENCFF001REG		ad/ENCFF752DDP.tsv	d/ENCFF173RJM.tsv	d/ENCFF742CVV.tsv
MCF7	Rep1	ENCFF000HQP	https://www.encodeproject.org/experiments/ENCSR000CPT/	https://www.encodeproject.org/files/ENCFF328CKZ/@@download/ENCFF328CKZ.tsv	https://www.encodeproject.org/files/ENCFF296ZWB/@@download/ENCFF296ZWB.tsv	https://www.encodeproject.org/files/ENCFF921PJP/@@download/ENCFF921PJP.tsv
		ENCFF000HQR				
	Rep2	ENCFF000HQQ		https://www.encodeproject.org/files/ENCFF930TQB/@@download/ENCFF930TQB.tsv	https://www.encodeproject.org/files/ENCFF030IUO/@@download/ENCFF030IUO.tsv	https://www.encodeproject.org/files/ENCFF967AOT/@@download/ENCFF967AOT.tsv
		ENCFF000HRH				

Supplementary Text Table 1 Description of ENCODE short read RNA-Seq data with downloading links

18. GO enrichment analysis on genes that share or disagree major isoforms between long and short read RNA-seq data

To test if differences in major isoform estimates between short and long reads occur in biologically relevant genes, we performed a GO enrichment analysis and compared the results to those for genes with concordant dominant isoforms between long and short read RNA-Seq data. We used enrichGO in the clusterProfiler R package [16] for each of the three GO classes: Biological Process (BP), Cellular Component (CC), and Molecular Function (MF). We then shortlisted significantly enriched GO terms where both the q-value and Benjamini-Hochberg adjusted p-value were significant (significance level: 0.05), and the gene ratio was greater than 0.01 for Molecular Function (MF) and Cellular Component (CC), and greater than 0.025 for Biological Process (BP). The results showed genes with discordant major isoforms fell into a large number of GO categories, which were similar to genes with concordant dominant isoforms between long and short read RNA-Seq data (Supplementary Text Fig. 21). This suggests that genes with observed differences in transcript expression estimates between long and short read RNA-Seq data are of broad functional relevance, impacting similar processes as genes with concordant isoforms.

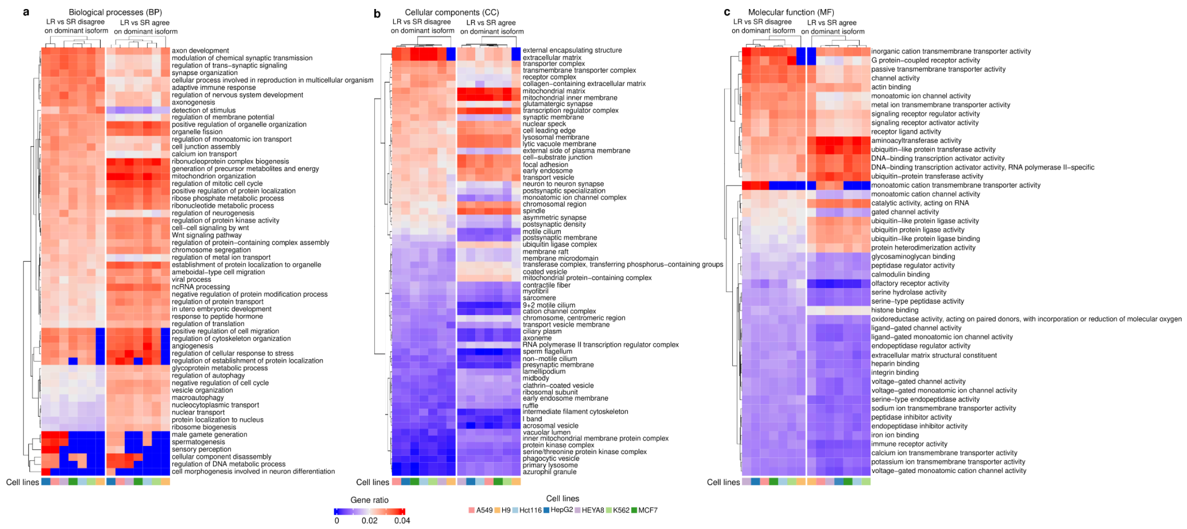

Supplementary Text Fig. 21 Genes with discordant or concordant dominant isoforms between long and short reads are enriched in similar GO categories

(a-c) Heatmaps showing the most significantly enriched GO terms in (a) Biological Processes categories with

gene ratio > 0.025, (b) Cellular Components (CC) categories with gene ratio > 0.01, and (c)Molecular Functions (MF) categories with gene ratio > 0.01, for genes with discordant or concordant dominant isoforms between long and short reads

19. Additional results from RT-qPCR and dPCR experiments

In our manuscript we report that short read and long read RNA-Seq identify different major isoforms for thousands of genes. By simulating read fragmentation from long read RNA-Seq data, we find that these results are likely an artefact of transcript expression quantification from short read RNA-Seq data, with long read RNA-Seq more robustly identifying major isoforms.

To provide independent experimental validation and control experiments for this claim, we have now selected 13 highly expressed genes from the MCF7 cell line where short read and long read RNA-Seq identified different major isoforms (discordant major isoforms), and quantified the expression of the long-read specific and short-read specific major isoforms using quantitative PCR (qPCR) and digital PCR (dPCR).

For each discordant major isoform pair, primers were designed to cover the unique sequences specific to each isoform. In some cases, where the short-read specific major isoform is a subset of the long-read specific major isoform (i.e., the splice junctions from the short-read specific major isoform are completely contained within the long-read specific major isoform), we compared the unique sequence from the long-read specific major isoform and the common sequence shared between the long-read and short-read specific major isoforms. We then performed both quantitative PCR (qPCR) and digital PCR (dPCR) on the developed assays. In qPCR experiments, we observed a very large log-fold-change in fluorescence density comparing long-read specific major isoforms to short-read specific major isoforms (Supplementary Text Fig. 22a). Also, we were able to validate all long-read specific major isoforms at very high concentration levels, while only a few short-read specific major isoforms were detected at much lower concentrations (Median ratio difference: 2417.6, Supplementary Text Fig. 22b-l).

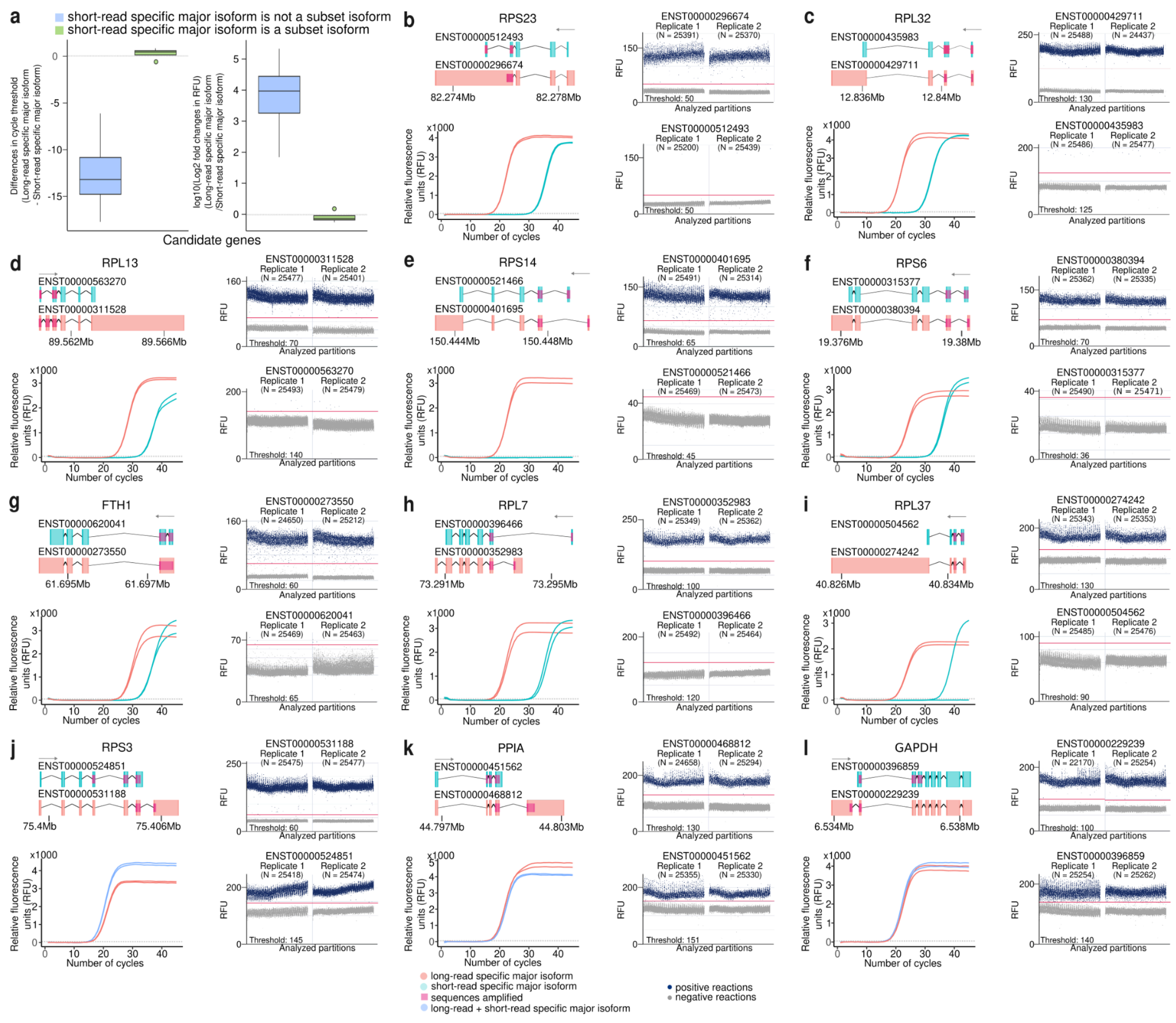

Supplementary Text Fig. 22 qPCR and dPCR results for selected candidates
(a) From left to right: Boxplots showing the median, upper and lower quartile, and 1.5 x interquartile ranges of cycle threshold the difference between long-read specific major isoform and short-read specific major isoform, and the log10-transformed log2-fold changes in relative fluorescence units between long-read specific major isoform and short-read specific major isoform (n=4 for short read specific major isoform is a subset isoform, n=9 for short read specific major isoform is not a subset isoform) **(b-l)** Top left: Genomic annotations for the long-read and short-read specific major isoforms and the sequences amplified for each isoform in RT-qPCR and dPCR experiments, for candidate genes where (b-i) short-read specific major isoform is not a subset isoform, or (j-l) short-read specific major isoform is a subset isoform. Bottom left: Line plots showing the relationship between the number of PCR cycles and the relative fluorescence units (RFU) in the RT-qPCR experiments, for the assays designed for the long-read and short-read specific major isoforms of , for candidate genes where (b-i) short-read specific major isoform is not a subset isoform, or (j-l) short-read specific major isoform is a subset isoform. Dotted grey line indicates the threshold defaulted at 50. Top right: The scatter plots showing the relative fluorescence units (RFU) in all analyzed partitions, for the assays designed for the long-read specific major isoforms of , for candidate genes where (b-i) short-read specific major isoform is not a subset isoform, or (j-l) short-read specific major isoform is a subset isoform. Dark blue indicates a positive reaction, and light grey indicates a negative reaction. Bottom right: The scatter plots showing the relative fluorescence units (RFU) in all analyzed partitions, for the assays designed for the short-read specific major isoforms of , for candidate genes where (b-i) short-read specific major isoform is not a subset isoform, or (j-l) short-read specific major isoform is a subset isoform. Dark blue indicates a positive reaction, and light grey indicates a negative reaction.

20. Alternative isoform switching analysis with edgeR

To check if different DTU detection methods give different results for alternative isoform switching analysis, we repeated the alternative isoform switch analysis with another

commonly used DTU detection method, edgeR [17,18] to compare the results obtained from DEXSeq followed by stageR.

We applied the same filtering steps before testing for DTU with DEXSeq and then repeated the analysis with edgeR. Here we performed two analyses: 1) to identify cell line specific DTU events by comparing one cell line against all other cell lines (cell line specific DTU events) as we have described in the main text, and 2) to identify cell line pairwise DTU events by comparing one cell line against another cell line (cell line pairwise DTU events) as an additional analysis.

As described in the main manuscript, we focused on major isoform switch events with at least 5 full-splice-match supporting reads in any of the samples involved in the comparisons. After which, we looked at significant DTU events defined as events with significant differential transcript expression (DTE) (Benjamini-Hochberg corrected p-value < 0.05), absolute log2 fold change being greater or equal to 2 and significant DTU (Benjamini-Hochberg corrected p-value < 0.05). Results were then compared with those obtained using our original approach described in the manuscript (DEXSeq followed by stageR).

In all 28 comparisons including both cell line-specific and cell line-pairwise comparisons, we found that edgeR reported significantly less number of events compared to DEXSeq (edgeR: median 10, IQR 7, 19.75 vs DEXSeq: median 46, IQR 37, 81; Mann-Whitney U test p < 0.0001), with on average 87% of the edgeR identified events also found by DEXSeq followed by stageR (median: 87%; IQR: 67%, 92%) (see Supplementary Fig. 6e). The average number of significant events identified by edgeR increased to 23 when we focused on significant DTE events instead, although still significantly less than the numbers reported by DEXSeq (edgeR: median 23, IQR 21, 37 vs DEXSeq: median 49, IQR 38, 81.75; Mann-Whitney U test p < 0.0001), with an average 85% of the edgeR identified events also found by DEXSeq (median: 85%; IQR: 66%, 92%) (see Supplementary Fig. 6f).

Overall, we found a large number of events identified by both methods, along with some events uniquely identified by each method. However, we believe that the stringent criteria we imposed on the candidate set, such as requiring full-splice-match read support and dominant expression, has helped to ensure the robustness of the findings from these methods.

21. Highly repetitive novel transcripts identified when different NDR applied and considering only retrotransposons

(1) Results reproduced using different NDR thresholds

To determine if the 10% false discovery rate used in this analysis (NDR = 0.1) is stringent enough, we now repeated the analysis using two other NDR thresholds: 0.05 and 0.15. We found consistent results across the different NDR thresholds, including significant enrichment of repeat overlapping and significantly lower expression of novel transcripts compared to annotated transcripts (see Supplementary Text Fig. 23a-b).

(2) Similar results when using only retrotransposons

To check if results remain valid when using only retrotransposons, we repeated the analysis using retrotransposons—namely, LINE, SINE, and LTR—along with potential retrotransposons (those marked with “?”) for all transcripts detected at NDR = 0.1. We found that results were consistent whether using only retrotransposons or all repeats. Novel transcripts, along with novel gene isoforms, were consistently more enriched in repeats compared to annotated transcripts with at least one full-splice-match read support (see Supplementary Text Fig. 23c). This suggests that retrotransposons are the dominant repeat type overlapping with these highly repetitive novel transcripts, which confirms our observations in Figure 6d. The findings were similar when examining novel transcripts filtered at varying NDR thresholds (0.05 and 0.15). Together, these results show that our claims are valid for both all repeats and when only retrotransposons are analysed.

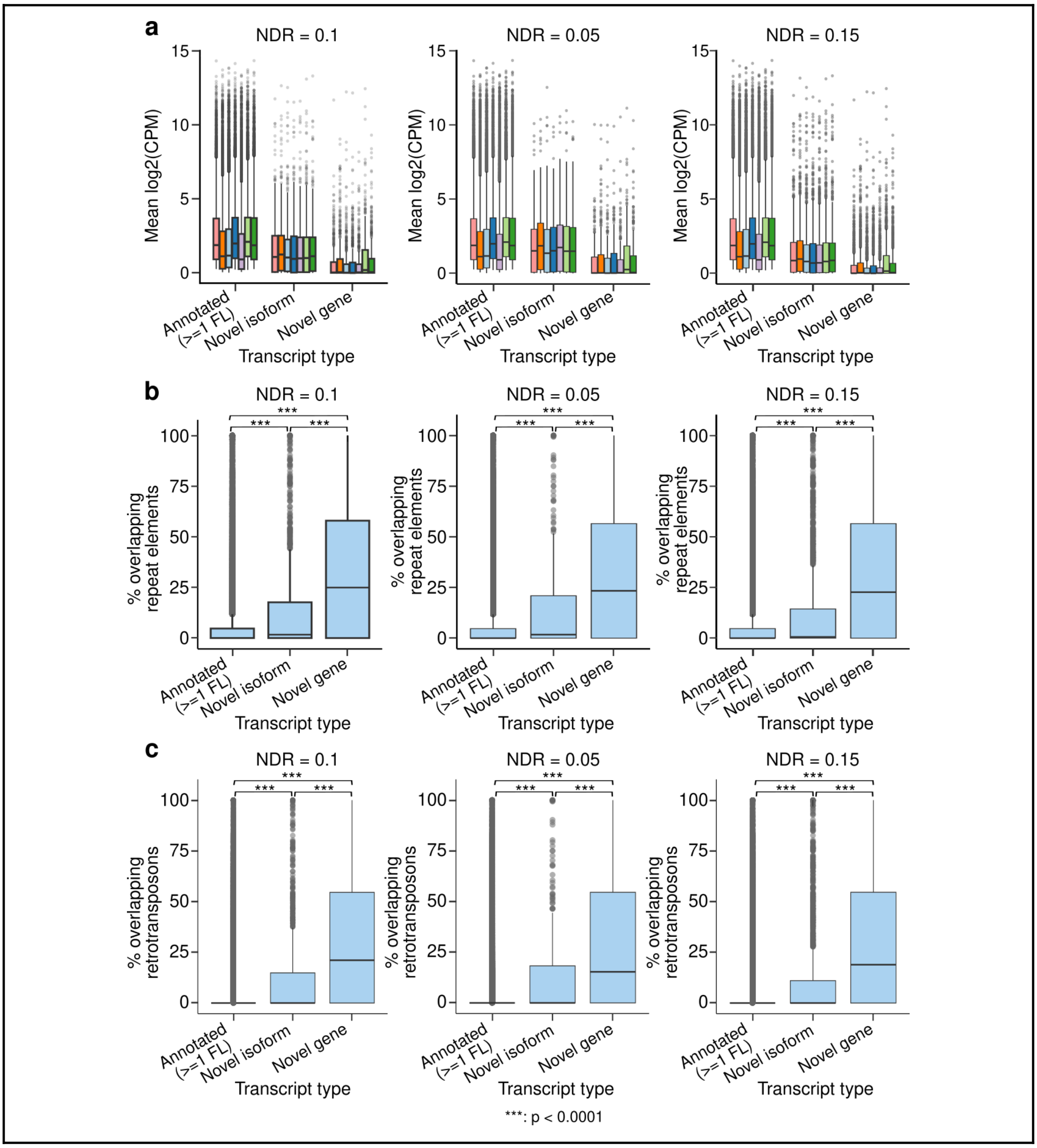

Supplementary Text Fig. 23 Novel transcripts had lower expression values, and higher repeat overlapping percentages across varying NDR thresholds
(a) Boxplots showing the median, upper and lower quartile, and 1.5 x interquartile ranges of the mean CPM for annotated transcripts with at least 1 full-length read support across samples (Annotated >=1 FL), all novel isoforms, and all novel gene isoforms, for 7 cell lines when NDR = 0.1 (n=35153,51858,45733,34588,44897,33692,37643 for A549, H9, Hct116, HepG2, HEYA8, K562, MCF7 respectively for Annotated >= 1 FL, n=908,617 for novel isoform and novel gene isoform specifically), 0.05 (n=35229,51969,45844,34656,44999,34020,37707 for A549, H9, Hct116, HepG2, HEYA8, K562, MCF7 respectively for Annotated >= 1 FL,n=295,293 for novel isoform and novel gene isoform specifically) and 0.15 (n=35063,51713,45609,34522,44765,33882,37561 for A549, H9, Hct116, HepG2, HEYA8, K562, MCF7 respectively for Annotated >= 1 FL,n=1952,1146 for novel isoform and novel gene isoform specifically, from left to right). **(b)** Boxplots showing the median, upper and lower quartile, and 1.5 x interquartile ranges of the percentage of exon sequence overlapping with repeat elements for annotated transcripts with at least 1 full-length read support across samples (Annotated >=1 FL), all novel isoforms, and all novel gene isoforms when NDR = 0.1 (n=74455, 908,617 for Annotated >= 1 FL, novel isoform, and novel gene isoform specifically), 0.05 (n=74571, 295, 293 for Annotated >= 1 FL, novel isoform and novel gene isoform specifically) and 0.15 (n=74310, 1952,1146 for Annotated >= 1 FL, novel isoform and novel gene isoform specifically, from left to right), with Bonferroni corrected two-sided Mann-Whitney U test p-values reported for the pairwise mean differences, three asterisks indicating p <= 0.0001 **(c)** Boxplots showing the median, upper and lower quartile, and 1.5 x interquartile ranges of the percentage of exon sequence overlapping with retrotransposons only for annotated transcripts with at least 1 full-length read support across samples (Annotated >=1 FL, n=74455), all novel isoforms, and all novel gene isoforms when NDR = 0.1 (n=74455,908,617 for Annotated >= 1 FL, novel isoform and novel gene isoform specifically), 0.05 (n=74571,295,293 for Annotated >= 1 FL, novel isoform and novel gene isoform specifically) and 0.15 (n=74310,1952,1146 for Annotated >= 1 FL, novel isoform and novel gene isoform specifically, from left to right), with Bonferroni corrected two-sided Mann-Whitney U test p-values reported for the pairwise mean differences, three asterisks indicating p <= 0.0001

22. New Hek293T sample sequenced using RNA004 kit

To highlight the novelty of m6A modification analysis using SG-NEx data, we have now added a new HEK293T sample generated using RNA004, the most recent direct RNA sequencing kit. The new sample generated a total of 12,470,494 reads, of which 9,160,370 (73.5%) are mappable. The average read length is 1,076 bp, with a 7% error rate and an average read quality score of 25.5.

We used this new sample to benchmark m6Anet and the recent basecaller Dorado [19], which allows for simultaneous basecalling and modification level estimation. We used two labelled datasets: 1) the m6ACE-seq labels in combination with miCLIP labels, which were previously used by Pratanwanich et al [20] and Hendra et al [21], and 2) GLORI-seq labels that was recently published [22].

We first compared the total number of potential m6A sites profiled in the new RNA004 sample against one replicate of HEK293T generated previously using the RNA002 kit. We found that about 7.7 times as many m6A sites were profiled in the RNA004 sample, suggesting that the increased sequencing depth has greatly enhanced the detection of m6A sites (Number of m6A sites in RNA004 sample vs that in RNA002 sample identified by m6Anet: 647,455 vs 84,189, Supplementary Text Fig. 24a).

When comparing Dorado and m6Anet on the new RNA004 sample, we found that Dorado additionally profiled 2,338,875 sites. This increase is potentially due to Dorado's ability to screen all sites with modified adenine, including non-DRACH motifs, which are usually ignored by other m6A detection methods. After excluding non-DRACH motif sites, we find that m6Anet and Dorado profiled the same m6A candidate sites (see Supplementary Text Fig. 24a).

We then compared the two m6A datasets (GLORI-Seq and m6ACE-Seq+ miCLIP-Seq) and found that GLORI-seq contained labels for significantly more sites (labels in GLORI-Seq only: 157,260 vs. labels in m6ACE-Seq+miCLIP only: 4,796). This difference remained high even when we included only labels for sites with a coverage-weighted mean modification rate across the two replicates being greater than or equal to 0.5 (labels in GLORI-Seq (>=0.5) only: 59,144 vs. labels in m6ACE-Seq+miCLIP only: 4,796), see Supplementary Text Fig. 24b.

We then compared the performance of Dorado and m6Anet on the new RNA004 sample, as well as m6Anet on the selected RNA002 sample as a control. We found that m6Anet achieved better performance than Dorado when all candidate sites were used for evaluation (Supplementary Text Fig. 24c). However, when we evaluated both methods only on sites that were profiled by both methods and which were covered in the RNA002 sample, we saw an improved performance for Dorado, in particular for sites with low modification rates (see Supplementary Text Fig. 24c-d). Together these results show that m6Anet and Dorado both identify a large number of sites that are confirmed by other experimental methods.

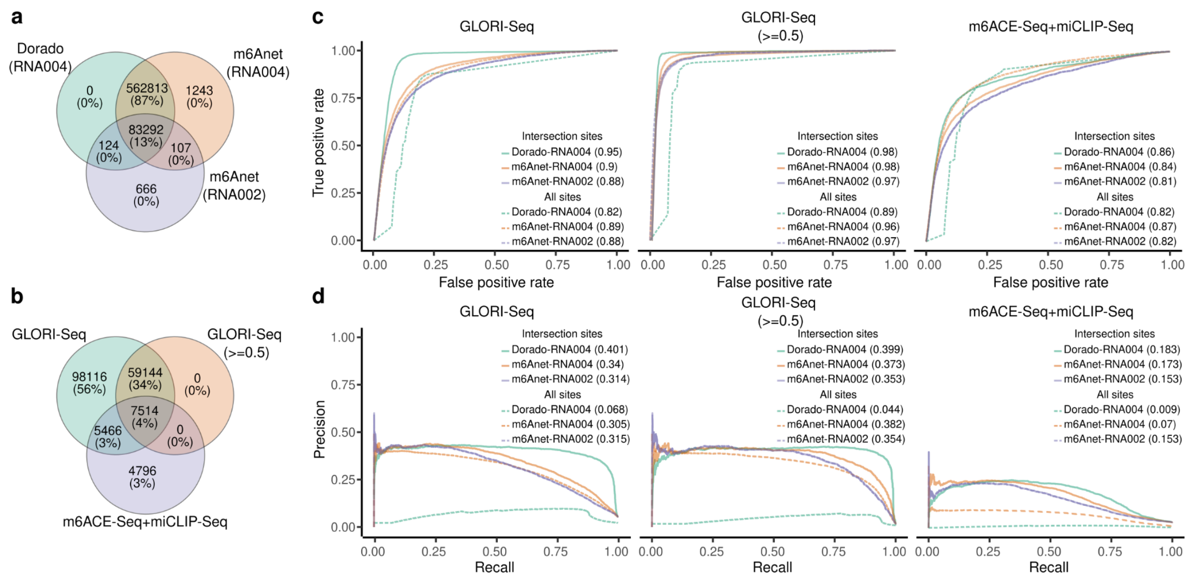

Supplementary Text Fig. 24 Dorado and m6Anet performed similarly for sample generated using RNA004

(a) Venn diagram showing the m6A sites profiled by Dorado, m6Anet, and m6Anet in RNA002 sample. Note here for Dorado, sites profiled by Dorado only were removed. **(b)** Venn diagram showing the overlap between labels defined in GLORI-Seq, GLORI-Seq (with coverage weighted mean modification rate >=0.5), and m6ACE-Seq + miCLIP-Seq **(c)** AUC curves showing the performance of Dorado, m6Anet in RNA004 sample and m6Anet in RNA002 sample, when using GLORI-Seq, GLORI-Seq (with coverage weighted mean modification rate >=0.5), and m6ACE-Seq + miCLIP-Seq labels. Solid lines represent results on sites shared between Dorado (RNA004), m6Anet (RNA004), and m6Anet (RNA002), and dotted lines represent results on all sites for each method. **(d)** PR curves showing the performance of Dorado, m6Anet in RNA004 sample and m6Anet in RNA002 sample, when using GLORI-Seq, GLORI-Seq (with coverage-weighted mean modification rate >=0.5), and m6ACE-Seq + miCLIP-Seq labels. Solid lines represent results on sites shared between Dorado (RNA004), m6Anet (RNA004), and m6Anet (RNA002), and dotted lines represent results on all sites for each method.

23. Filtering potential RT and intra-priming artefacts with SQANTI3

To determine if RTS and intra-priming artefacts are impacting the identification of highly repetitive novel transcripts, we performed SQANTI3 [23] on the novel annotations generated by Bambu, using all SG-NEx samples with an NDR threshold of 0.1. We found that out of all 1,635 novel transcripts, 94 (5.7%) are classified as RTS artefacts (see Supplementary Text Fig. 25a). Among these, only 2 overlap by more than 80% with repeat elements (2 out of 140, 1.4%), suggesting that most highly repetitive transcripts are not caused by RTS artefacts (Supplementary Text Fig. 25a). In terms of potential intra-priming artefacts, 12 (0.7%) of the novel transcripts have polyA tails comprising more than 60% of total A bases (following the default threshold as recommended in SQANTI3, Supplementary Text Fig. 25a). Similarly, 2 of these transcripts overlap by more than 80% with repeat elements (2 out of 140, 1.4%), showing that most highly repetitive transcripts are not caused by intra-priming artefacts (Supplementary Text Fig. 25a). Both results suggest that, at an NDR threshold of 0.1, most of the highly repetitive novel transcripts are neither RTS nor intra-priming artefacts. We have now included SQANTI3 QC in our analysis and removed novel transcripts identified as potential RTS and intra-priming artefacts.

24. Impact of read quality filtering on highly repetitive novel transcripts

To investigate the impact of read quality filtering on the identification of repetitive transcripts, we also removed reads with Phred quality scores less than 7 (< Q7), which filtered out, on average, 5% (IQR: 2% to 5.4%) of reads. We then analysed RTS and intra-priming artefacts again with SQANTI3 on the novel transcripts identified at NDR = 0.1 using the reads that passed the Q7 filter. We found that a similar number of RTS or intra-priming artefacts were detected before and after Q7 filtering (number of potential RTS artefacts before vs. after Q7 filtering: 94 vs. 96; number of potential intra-priming artefacts before vs. after Q7 filtering: 12 vs. 9). All 4 highly repetitive RTS or intra-priming artefacts were still identified (see Supplementary Text Fig. 25b). These results demonstrate that our findings regarding highly repetitive novel transcripts are robust, even when read quality filtering is applied.

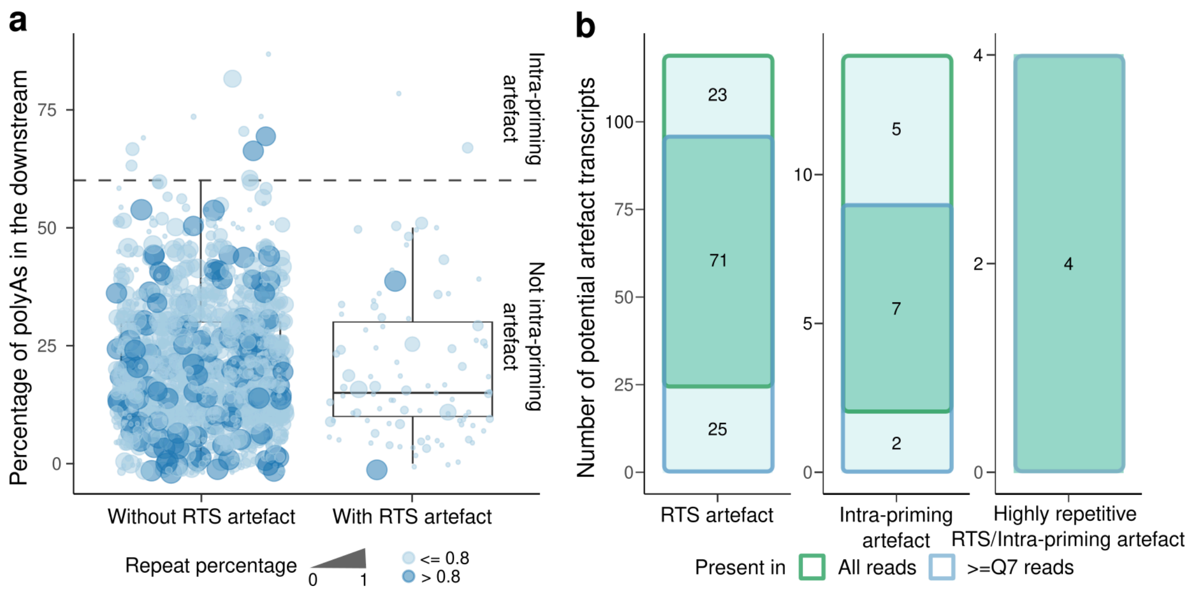

Supplementary Text Fig. 25 Few novel transcripts classified as potential RTS or intra-priming (polyA percentage > 60%) artefacts
(a) Boxplots showing the median, upper and lower quartile, and 1.5 x interquartile ranges of the percentage of polyAs in the downstream of potentially RTS artefact novel transcripts (n=94) vs non-RTS artefact novel transcripts (n=1541), complementary to Figure 6a (NDR =0.1). The point size indicated the percentage of overlapping with repeat elements, with dark blue representing repeat overlapping being greater than 80%. **(b)** Number of potential RTS, Intra-priming, or highly repetitive RTS/Intra-priming artefact transcripts found when using all reads or reads passing Q7 filter.

25. Summary of spike-in set characteristics

To clarify the metrics used for each set of spike-in RNAs, we have now included a table describing each set in terms of the number of genes and isoforms, transcript length, and concentration variety (see Supplementary Text Table 2).

	**Sequin (MixA V1)**	**Sequin (MixA V2)**	**SIRV (E0)**	**SIRV (E2)**	**ERCC**	**Long SIRV**
Total number of genes	78		7		92	5
Total number of isoforms	164		69		92	15
*Number of isoforms per gene**	2<br>(1,4)		8<br>(6,18)		1<br>(1,1)	3<br>(3,3)
*Number of exons per isoform**	6<br>(1,36)		4(1,18)		1<br>(1,1)	1<br>(1,1)
*Isoform length**	1045<br>(283, 6943)		783<br>(161, 2498)		978<br>(256,2014)	8000<br>(3997, 12029)
*Intron length**	1793<br>(71, 544891)		283<br>(21, 110785)		0	0
*Exon length**	126<br>(15,4414)		125<br>(9,2473)		978<br>(256, 2014)	8000<br>(3997, 12029)
Number of unique concentration values	81	117	1	4	22	1

*Concentration values**	13.43 (0.004,30000)	4.23 (0.004,1691.89)	60 (60,60)	625 (31.25,4000)	1.1 (0.001,1500)	60 (60,60)

*: median (min, max)

Supplementary Text Table 2 Characteristics of spike-in sets included in SG-NEx data resource

26. Long read enables profiling of full-length fusion transcripts

This not only identifies transcripts with alternative breakpoint junctions but also enables the discovery of associated splicing events away from the breakpoint that are specific to the fusion transcripts (Supplementary Fig. 9a). The ability to profile complete fusion transcripts is further demonstrated for the well-described breakpoint that leads to the fusion gene *TXLNG-SYAP1*. Using the SG-NEx data, we identified a previously described *TXLNG* fusion transcript that uses an alternative exon before the breakpoint on chromosome 22, which is supported by full-splice-match reads, and validated by PCR [24](Supplementary Fig. 9b).

Supplementary Notes

1. Analysis of alternative isoform expression

(1) Alternative isoforms and splicing events in the SG-NEx cell lines

To characterise the general isoform usage, we compared the major isoform with minor isoforms within each cell line and studied the usage of promoters, last exons, exon skipping, intron retention, and alternative splicing at 5' or 3' ends.

Isoform switching events can be generally classified into three categories: alternative promoters (alternative first exons), alternative transcription end sites usage (alternative last exons), or alternative splicing events. For alternative promoters, we compared the first exons and flagged the event as alternative promoter if the first exons were non-overlapping. Similarly, we flagged the event as alternative last exons if the last exons were non-overlapping. Alternative splicing can be classified as three different types: (1) alternative splicing at 3' or 5' ends; (2) exon skipping; or (3) intron retention. An exon skipping event was identified by checking for the containment of the exon ranges of the reference major isoform within the junction ranges of the alternative dominant isoforms. Similarly, an intron retention event was identified by checking for the containment of the junction ranges of the reference major isoform within the exon ranges of the alternative isoforms. Alternative 5' or 3' splicing was identified as present if the exons are spliced differently at their 3' or 5' ends.

To include alternative isoforms in this analysis, we required that the major isoform has an average full-splice-match normalised read count >= 2 across replicates from the respective cell lines. Here we calculated the normalised full-splice-match read count for each transcript as the number of full-splice-match reads divided by the total number of full-splice-match reads in the sample, multiplied with 1 million (corresponding to the definition of CPM for total counts). Furthermore, the reference and the alternative isoforms had to be expressed with average expression >= 2 CPM across replicates from the respective cell lines, and the average expression for each isoform had to contribute to at least 5% of the total average expression for this gene in the respective cell line. For the analysis of alternative splicing events with Salmon, NanoCount and Bambu (without full-splice-match read support requirement), only the expression filters were applied.

(2) Differential transcript usage

For differential gene expression analysis, we used DESeq2 [10]. For differential transcript/promoter/transcription end site usage, we used DEXSeq [25]. In the filtering step, we used DRIMSeq [26] to filter genes with no more than 10 reads aggregated across at least 12 samples (the largest sample size for one of the cell line group), and isoforms with no more than 10 reads across at least 9 samples (the smallest sample size for one of the cell line group). The minimum sample-to-feature ratio was set as 0.1 by default. Although the above filtering criterion could identify important differentially used isoforms across cell lines, it also identified negative events where genes were only expressed in non-reference cell lines as the sample size of the non-reference cell lines are larger than the required sample size. To fix this issue, we implemented an additional filtering criterion, where we removed genes without any expression in the reference cell line. For the analysis, we adjusted for different protocols by including the protocol as a covariate in the model. Differential transcript usage

was tested with a two-stage testing (DEXSeq followed by stageR), where a transcript is identified as differentially used only if it has an adjusted DEXSeq p-value < 0.05 and also gene and transcript p-values obtained from stageR < 0.05. After identifying the genes and transcripts that contributed to the isoform switching events, we calculated whether the contributing transcripts were dominant isoforms of the reference cell line by finding the most highly expressed isoform within each gene using the average expression levels, which was then used to decide if it was a dominant isoform switching event.

For major isoform switching events, we compared the isoform from the reference cell line (reference major isoform) and the major isoforms from other cell lines (alternative dominant isoform). We then determined the isoform switching types. We kept isoform switching events with at least 5 full-splice-match reads supported in any of the samples for both the reference dominant isoform and the alternative dominant isoform. For complete dominant isoform switching events, we also compared the isoform from the reference cell line to the minor isoforms from other cell lines.

We used estimates obtained from Bambu for the above analysis. We also repeated the analysis with estimates obtained from NanoCount and Salmon, and DTU event detection with edgeR (Supplementary Fig. 6).

2. Novel transcripts and repeat enrichment analysis

(1) Analysis of transcript discovery threshold

Transcript discovery was done with Bambu on all samples from the core and extended SG-NEx data set simultaneously, using the direct RNA, direct cDNA, and PCR-cDNA protocols. Bambu returns a set of extended annotations, transcript and gene abundance estimates that are matched for each sample.

We first investigated the novel isoform from the annotated genes (novel isoform) and novel isoform from the novel genes (novel gene) counts when we applied Bambu with different discovery sensitivity parameters (NDR: 0.025 to 0.25 at a step of 0.025) to understand the impact of transcript discovery sensitivity on novel transcript discovery. We extended the analysis to a larger range of NDR from 0.1 to 1 at a step of 0.1 (Supplementary Figure 7). For this analysis, we used the extended annotation set obtained at an NDR of 0.1 using all SG-NEx samples and filtered out potential reverse transcription switch (RTS) and intra-priming artefacts identified by SQANTI3, version 5.2 [23]. Transcript candidates were classified as potential RTS artefacts if the RTS_stage status is TRUE, and as intra-priming artefacts if perc_A_downstream_TTS is scored higher than 60, following the recommended threshold in SQANTI3. As a comparison, we also repeated the analysis with estimates obtained from StringTie2 with and without single-exon novel isoforms included. We compared the results with the extended annotations set obtained at NDR = 0.1 using the 6 PacBio samples in the SG-NEx data resource.

(2) Analysis of expression for novel isoforms

We compared the expression levels for novel isoforms from annotated genes, and novel isoforms from novel genes detected at NDR = 0.1, against annotated isoforms with at least 1 full-splice-match read support.

(3) Overlap of transcripts with repetitive elements

To estimate the overlap between transcripts and repeat elements, we used the RepeatMasker annotations, and calculated the number of bases in each exon that overlap with any annotated repeat class. We distinguished between annotated exons and novel exons based on whether the exon overlapping with the reference annotations. We then calculated the percentage of repetitive sequence for each isoform, and in addition for the annotated and novel part of each isoform separately.

A two-sided Mann-Whitney U test was applied to test the overlapping percentages between annotated and novel transcripts.

(4) Analysis of multi-mapped reads and their impact on transcript discovery

The above analysis was first conducted with all primary alignments from the minimap2 default alignment setting (allowing up to 5 multiple alignments per reads). To mitigate the impact of multiple alignments, we repeated the analysis with reads having only one alignment (unique alignment), i.e., only reads that have a single alignment were kept and reprocessed with Bambu for transcript discovery. By doing so, on average, 68.8% reads were kept for this analysis.

(5) RT-PCR validation of the novel transcripts

Among all novel transcript candidates that were expressed in MCF7 cell line, we shortlisted 9 candidates that were top expressed novel isoforms of annotated protein coding genes, with some overlapping with repeat elements. Up to 5 µg of total RNA of breast cancer cell lines MCF7 was reverse-transcribed using SuperScript™ III First-Strand Synthesis System (Invitrogen) according to the manufacturers' instructions. Primers were designed using Primer3 (https://bioinfo.ut.ee/primer3-0.4.0/) based on the putative novel transcript sequence predicted by Bambu. For one of the 9 candidates (BambuTx193), two unique junctions were targeted with two different sets of primers. PCR was done with JumpStart RedAccuTaq LA DNA Polymerase (Merck) with the following thermo cycling conditions: 98 °C for 30 s, (94 °C for 15 s, 58 °C for 20 s and 68 °C for 2 min) ×15 and (94 °C for 15 s, 55 °C for 20 s and 68 °C for 2 min) ×20 and 68 °C for 5 min. PCR products were purified with DNA clean & Concentrator kits (Zymo Research) and were analyzed with D1000 ScreenTape assay (Agilent). The PCR products with the expected size were sent for Sanger Sequencing (1st Base).

3. Fusion gene analysis

(1) Fusion calling method

For each cell line in the SG-NEx core dataset, reads from replicates were combined and analysed with the JAFFAL pipeline of JAFFA (version 2.0,

https://github.com/Oshlack/JAFFA) [27]. JAFFAL detects fusions in long read data by aligning reads to a reference transcriptome (Gencode version 22) using Minimap2. Reads aligning to multiple genes were flagged as candidate fusion reads and aligned to the human reference genome, hg38, using Minimap2. Fusions with breakpoints within 300kbp of each other and the 5' and 3' genes in transcriptional order, were consistent with read-through transcription and were removed. Fusions involving genes on the mitochondrial chromosome or with low read support (<3) were also removed. Four fusions found across multiple cell lines were likely to be false and hence only those unique to a cell line were retained. Fusions previously validated in the same cell lines were identified from a literature search (Supplementary Table 8) [28–37]. Fusions were identified in the Illumina 150bp paired-end data by running JAFFA's Direct pipeline on each replicate of each core cell line.

(2) Fusion alignment

With the identified fusion gene candidates, we first created a fusion genome that combined the reference genome sequences of the two fusion genes into a single fusion gene chromosome for each detected fusion. We then aligned reads overlapping with any of the candidate genes in the fusion gene set to the fusion genome sequences using minimap2 with parameters "-ax splice -G2200k -N 5 --sam-hit-only".

(3) Fusion transcript discovery and quantification

We ran Bambu on those fusion genome alignments to discover fusion transcripts and quantify the fusion transcript abundance using all samples in the core SG-NEx data. We used a NDR threshold of 1 and required a minimum read count of 1 in at least 1 sample for a fusion transcript to be included in quantification. To quantify 5' genes, we aggregated full-splice-match read support for all transcripts that fell only within the 5' gene range. Similarly, we aggregated full-splice-match support for the 3' gene. Full-splice-match read counts for transcripts that overlapped with the fusion gene range but not contained only within 5' or 3' gene ranges were aggregated as the full-splice-match read support for the fusion gene.

(4) Fusion validation

From all fusion gene candidates identified in MCF7 cell line, we shortlisted 12 candidates by filtering based on their read count support and manual validation in the IGV browser. We then designed PCR primers targeting the breakpoints for these 12 candidates and performed RT-PCR and Sanger sequencing [38], as described above in RT-PCR validation of the novel transcripts.

4. m6A modification analysis

(1) Read segmentation

For the analysis of m6A RNA modifications, reads were aligned to the transcriptome (please refer to "Read Alignment" for details). For each direct RNA-Seq sample in the SG-NEx core

dataset, the raw current signal from each read was aligned to the reference transcript sequence using nanopolish eventalign [39].

(2) Identification of m6A using m6Anet

The reference-aligned signal events were then processed and analysed with m6Anet [21] (https://github.com/GoekeLab/m6anet), which predicts the probability of DRACH motifs being modified. All analyses were done on DRACH positions with support by ≥ 50 reads (Supplementary Table 11). Unless otherwise specified, we defined DRACH motifs with an m6Anet-predicted m6A probability ≥ 0.9 as modified (m6A sites) and all other positions as unmodified.

(3) Metagene and motif analysis

The relative position of the predicted m6A sites (all, probability > 0.5, or probability > 0.9) and the density at each relative position were calculated with Python packages seaborn and matplotlib (Supplementary Fig. 10b). The nucleotide content of modified and unmodified m6A sites was visualized with the ggseqlogo R package [40] (Supplementary Fig. 10c).

(4) Comparison of m6A sites across samples

To compare m6A sites across different direct RNA-Seq samples, we calculated a pairwise enrichment score, defined as the odds ratio (OR) of the tested two samples having similar m6A sites (p>0.9) for each pair of the samples and we visualized the enrichment scores with the ComplexHeatmap R package [41](Fig. 6f). We also calculated the pairwise Pearson correlation of the modification probability for all m6A sites across these samples and visualized the results with the ComplexHeatmap R package [41](Supplementary Fig. 10e).

To calculate the number of cell lines in which each m6A site occurs, we first calculated the read-count weighted average of predicted m6A probability at each site with at least 50 reads from all replicates of the same cell line. We then counted the number of cell lines in which each m6A site was detected (based on the recommended threshold of p=0.9), with two scenarios: (1) using only sites which were covered by at least 50 reads in all cell lines (i.e. sites from transcripts which were expressed in all cell lies), and (2) using sites which were covered by at least 50 reads in at least one cell line (i.e. sites from transcripts which were expressed in at least one cell line) (Fig. 6g).

(5) Ranking of highly modified genes in the SG-NEx data

In order to rank genes by the level of m6A modifications, we first calculated the read-count weighted m6A probability across all samples for each m6A site. We then estimated the number of m6A sites for each gene as the number of m6A sites with read-count weighted m6A probability > 0.9 (Supplementary Fig. 10e). The *MYC* gene's m6ACE-Seq-detected m6A sites, cell-line-averaged m6Anet-predicted m6A probability, and cell-line-averaged coverage were plotted using the R packages sushi and ggplot2 (Fig. 6h). All m6A positions of the 50 most highly modified genes across direct RNA samples from the 7 core cell lines were plotted as a heatmap with a color gradient indicating the predicted m6A probability

using the gplots R package with default parameters for clustering the m6A positions and the cell lines (Supplementary Fig. 10f).

References

1. Kodzius, R. *et al.* CAGE: cap analysis of gene expression. *Nature Methods* **3**, 211–222 (2006).
2. Pardo-Palacios, F. J. *et al.* Systematic assessment of long-read RNA-seq methods for transcript identification and quantification. *Nature Methods* **21**, 1349–1363 (2024).
3. Tardaguila, M. *et al.* SQANTI: extensive characterization of long-read transcript sequences for quality control in full-length transcriptome identification and quantification. *Genome Res.* **28**, 396–411 (2018).
4. Prawer, Y. D. J., Gleeson, J., De Paoli-Iseppi, R. & Clark, M. B. Pervasive effects of RNA degradation on Nanopore direct RNA sequencing. *NAR Genom Bioinform* **5**, lqad060 (2023).
5. Derti, A. *et al.* A quantitative atlas of polyadenylation in five mammals. *Genome Res.* **22**, 1173–1183 (2012).
6. Lin, Y. *et al.* An in-depth map of polyadenylation sites in cancer. *Nucleic Acids Res* **40**, 8460–8471 (2012).
7. Ye, C., Long, Y., Ji, G., Li, Q. Q. & Wu, X. APAtrap: identification and quantification of alternative polyadenylation sites from RNA-seq data. *Bioinformatics* **34**, 1841–1849 (2018).
8. Niazi, A. M., Krause, M. & Valen, E. Transcript Isoform-Specific Estimation of Poly(A) Tail Length by Nanopore Sequencing of Native RNA. *RNA Bioinformatics* 543–567 (2021) doi:10.1007/978-1-0716-1307-8_30.
9. GitHub - epi2me-labs/pychopper: cDNA read preprocessing. *GitHub* https://github.com/epi2me-labs/pychopper.
10. Love, M. I., Huber, W. & Anders, S. Moderated estimation of fold change and dispersion for RNA-seq data with DESeq2. *Genome Biol.* **15**, 550 (2014).
11. Love, M. I., Anders, S., Kim, V. & Huber, W. RNA-Seq workflow: gene-level exploratory analysis and differential expression. *F1000Res* **4**, 1070 (2015).
12. Love, M. I., Soneson, C. & Patro, R. Swimming downstream: statistical analysis of differential transcript usage following Salmon quantification. *F1000Res.* **7**, 952 (2018).
13. Moore, J. E. *et al.* Expanded encyclopaedias of DNA elements in the human and mouse genomes. *Nature* **583**, 699–710 (2020).
14. Bray, N. L., Pimentel, H., Melsted, P. & Pachter, L. Near-optimal probabilistic RNA-seq quantification. *Nature Biotechnology* **34**, 525–527 (2016).

15. Li, B. & Dewey, C. N. RSEM: accurate transcript quantification from RNA-Seq data with or without a reference genome. *BMC Bioinformatics* **12**, 1–16 (2011).
16. Yu, G., Wang, L.-G., Han, Y. & He, Q.-Y. clusterProfiler: an R Package for Comparing Biological Themes Among Gene Clusters. (2012) doi:10.1089/omi.2011.0118.
17. Robinson, M. D., McCarthy, D. J. & Smyth, G. K. edgeR: a Bioconductor package for differential expression analysis of digital gene expression data. *Bioinformatics* **26**, 139–140 (2010).
18. Chen, Y., Chen, L., Lun, A. T. L., Baldoni, P. L. & Smyth, G. K. edgeR 4.0: powerful differential analysis of sequencing data with expanded functionality and improved support for small counts and larger datasets. *bioRxiv* 2024.01.21.576131 (2024) doi:10.1101/2024.01.21.576131.
19. Oxford Nanopore Technologies PLC. *Dorado*. (2024).
20. Pratanwanich, P. N. *et al.* Identification of differential RNA modifications from nanopore direct RNA sequencing with xPore. *Nature Biotechnology* **39**, 1394–1402 (2021).
21. Hendra, C. *et al.* Detection of m6A from direct RNA sequencing using a Multiple Instance Learning framework. *bioRxiv* 2021.09.20.461055 (2021) doi:10.1101/2021.09.20.461055.
22. Liu, C. *et al.* Absolute quantification of single-base m6A methylation in the mammalian transcriptome using GLORI. *Nature Biotechnology* **41**, 355–366 (2022).
23. Pardo-Palacios, F. J. *et al.* SQANTI3: curation of long-read transcriptomes for accurate identification of known and novel isoforms. *Nat. Methods* **21**, 793–797 (2024).
24. Chen, Y. *et al.* Gene Fusion Detection and Characterization in Long-Read Cancer Transcriptome Sequencing Data with FusionSeeker. *Cancer Res* **83**, 28–33 (2023).
25. Reyes, A. *et al.* Drift and conservation of differential exon usage across tissues in primate species. *Proc. Natl. Acad. Sci. U. S. A.* **110**, 15377–15382 (2013).
26. Nowicka, M. & Robinson, M. D. DRIMSeq: a Dirichlet-multinomial framework for multivariate count outcomes in genomics. *F1000Res.* **5**, 1356 (2016).
27. Davidson, N. M., Majewski, I. J. & Oshlack, A. JAFFA: High sensitivity transcriptome-focused fusion gene detection. *Genome Med.* **7**, 43 (2015).
28. Edgren, H. *et al.* Identification of fusion genes in breast cancer by paired-end RNA-sequencing. *Genome Biol.* **12**, R6 (2011).
29. Kangaspeska, S. *et al.* Reanalysis of RNA-sequencing data reveals several additional fusion genes with multiple isoforms. *PLoS One* **7**, e48745 (2012).

30. Sakarya, O. *et al.* RNA-Seq mapping and detection of gene fusions with a suffix array algorithm. *PLoS Comput. Biol.* **8**, e1002464 (2012).
31. Maher, C. A. *et al.* Chimeric transcript discovery by paired-end transcriptome sequencing. *Proc. Natl. Acad. Sci. U. S. A.* **106**, 12353–12358 (2009).
32. Inaki, K. *et al.* Transcriptional consequences of genomic structural aberrations in breast cancer. *Genome Res.* **21**, 676–687 (2011).
33. Asmann, Y. W. *et al.* A novel bioinformatics pipeline for identification and characterization of fusion transcripts in breast cancer and normal cell lines. *Nucleic Acids Res.* **39**, e100 (2011).
34. Francis, R. W. *et al.* FusionFinder: a software tool to identify expressed gene fusion candidates from RNA-Seq data. *PLoS One* **7**, e39987 (2012).
35. Nome, T. *et al.* Common fusion transcripts identified in colorectal cancer cell lines by high-throughput RNA sequencing. *Transl. Oncol.* **6**, 546–553 (2013).
36. Nome, T. *et al.* High frequency of fusion transcripts involving TCF7L2 in colorectal cancer: novel fusion partner and splice variants. *PLoS One* **9**, e91264 (2014).
37. Vellichirammal, N. N. *et al.* Pan-Cancer Analysis Reveals the Diverse Landscape of Novel Sense and Antisense Fusion Transcripts. *Mol. Ther. Nucleic Acids* **19**, 1379–1398 (2020).
38. Peng, H. *et al.* Development and Validation of an RNA Sequencing Assay for Gene Fusion Detection in Formalin-Fixed, Paraffin-Embedded Tumors. *J. Mol. Diagn.* **23**, 223–233 (2021).
39. Loman, N. J., Quick, J. & Simpson, J. T. A complete bacterial genome assembled de novo using only nanopore sequencing data. *Nat. Methods* **12**, 733–735 (2015).
40. Wagih, O. ggseqlogo: a versatile R package for drawing sequence logos. *Bioinformatics* **33**, 3645–3647 (2017).
41. Gu, Z., Eils, R. & Schlesner, M. Complex heatmaps reveal patterns and correlations in multidimensional genomic data. *Bioinformatics* **32**, 2847–2849 (2016).
