# Supplementary material for: A systematic benchmark of Nanopore long-read RNA sequencing for transcript-level analysis in human cell lines
Source: Nat Methods. 2025 Mar 13;22(4):801–12. doi: 10.1038/s41592-025-02623-4 (PMC11978509; doi:10.1038/s41592-025-02623-4)
Supplement: Supplementary file 2 — Reporting Summary [file 41592_2025_2623_MOESM2_ESM.pdf]

nature research

Corresponding author(s): Jonathan Göke

Last updated by author(s): Nov 26, 2024

Reporting Summary

Nature Research wishes to improve the reproducibility of the work that we publish. This form provides structure for consistency and transparency in reporting. For further information on Nature Research policies, see our Editorial Policies and the Editorial Policy Checklist.

Statistics

For all statistical analyses, confirm that the following items are present in the figure legend, table legend, main text, or Methods section.

n/a	Confirmed	
☐	☒	The exact sample size (*n*) for each experimental group/condition, given as a discrete number and unit of measurement
☐	☒	A statement on whether measurements were taken from distinct samples or whether the same sample was measured repeatedly
☐	☒	The statistical test(s) used AND whether they are one- or two-sided<br>*Only common tests should be described solely by name; describe more complex techniques in the Methods section.*
☒	☐	A description of all covariates tested
☐	☒	A description of any assumptions or corrections, such as tests of normality and adjustment for multiple comparisons
☐	☒	A full description of the statistical parameters including central tendency (e.g. means) or other basic estimates (e.g. regression coefficient) AND variation (e.g. standard deviation) or associated estimates of uncertainty (e.g. confidence intervals)
☐	☒	For null hypothesis testing, the test statistic (e.g. F, t, r) with confidence intervals, effect sizes, degrees of freedom and P value noted<br>*Give P values as exact values whenever suitable.*
☒	☐	For Bayesian analysis, information on the choice of priors and Markov chain Monte Carlo settings
☒	☐	For hierarchical and complex designs, identification of the appropriate level for tests and full reporting of outcomes
☐	☒	Estimates of effect sizes (e.g. Cohen's *d*, Pearson's *r*), indicating how they were calculated

Our web collection on statistics for biologists contains articles on many of the points above.

Software and code

Policy information about availability of computer code

Data collection	Cell growth and RNA extraction protocols are described in Supplementary Table 1.
Data analysis	Sequencing libraries were prepared using the Nanopore direct RNA, direct cDNA, and PCR cDNA kits, short read sequencing (Illumina paired end 150 bp), and PacBio long read SMRTcell. Details and deviations are described in Supplementary Table 1. Sequencing runs were performed using MinION/GridION (FLO-MIN106/106D/107), or PromethION (FLO-PRO001/002) (Oxford Nanopore Technologies), Illumina HiSeq 4000 system, and PacBio Sequel II system(Supplementary Table 1). nf-core/nanoseq is a streamlined, community curated pipeline for Nanopore Sequencing data processing and analysis: https://nf-co.re/nanoseq The code used for this manuscript is deposited in github repository and can be accessed here: https://github.com/GoekeLab/sg-nex-data/tree/master/manuscript.

For manuscripts utilizing custom algorithms or software that are central to the research but not yet described in published literature, software must be made available to editors and reviewers. We strongly encourage code deposition in a community repository (e.g. GitHub). See the Nature Research guidelines for submitting code & software for further information.

Data

Policy information about availability of data

All manuscripts must include a data availability statement. This statement should provide the following information, where applicable:

- Accession codes, unique identifiers, or web links for publicly available datasets
- A list of figures that have associated raw data
- A description of any restrictions on data availability

The SG-NEx data is available at: https://github.com/GoekeLab/sg-nex-data. All data is deposited in ENA (https://www.ebi.ac.uk/ena/browser/view/PRJEB44348). Fast5 (BLOW5), fastq, bam, and additional processed data is made available for fast compute optimised access on S3 through the AWS open data sponsorship (http://sg-nex-data.s3-website-ap-southeast-1.amazonaws.com/ and http://sg-nex-data-blow5.s3-website-ap-southeast-1.amazonaws.com/) . We also downloaded

the datasets from the ENCODE portal (Sloan et al. 2016) (https://www.encodeproject.org/) with the following identifiers: ENCSR000CON, ENCSR000CWM, ENCSR000CPE, ENCSR000AEM, ENCSR000CPT (see Supplementary Text Table 1 for detailed download links).

Field-specific reporting

Please select the one below that is the best fit for your research. If you are not sure, read the appropriate sections before making your selection.

☒ Life sciences ☐ Behavioural & social sciences ☐ Ecological, evolutionary & environmental sciences

For a reference copy of the document with all sections, see nature.com/documents/nr-reporting-summary-flat.pdf

Life sciences study design

All studies must disclose on these points even when the disclosure is negative.

Sample size	To perform RNA-Seq differential expression analysis, we acquired a minimum number of at least 3 biological replicates for each protocol run from each cell line, for differential expression analysis (Schurch et al, 2016).
Data exclusions	Sequencing runs with less than 400,000 reads were excluded as these runs generally show low quality compared to the other runs and may produce unreliable results.
Replication	RT-PCR experiments to confirm novel transcripts were conducted once, with external validation from recent Gencode/Ensembl release. RT-qPCR experiment results to confirm long-read specific major isoforms were confirmed with dPCR experiments.
Randomization	Batch effects adjustment and covariate adjusted analysis is performed to control for potential confounding effects of sequencing platforms and batches.
Blinding	Blinding is not relevant to the study as no allocation is needed for data during data collection and analysis, as they are all predefined by the sequencing technologies and cell line types.

Reporting for specific materials, systems and methods

We require information from authors about some types of materials, experimental systems and methods used in many studies. Here, indicate whether each material, system or method listed is relevant to your study. If you are not sure if a list item applies to your research, read the appropriate section before selecting a response.

Materials & experimental systems

n/a	Involved in the study	
☒	☐	Antibodies
☐	☒	Eukaryotic cell lines
☒	☐	Palaeontology and archaeology
☒	☐	Animals and other organisms
☒	☐	Human research participants
☒	☐	Clinical data
☒	☐	Dual use research of concern

Methods

n/a	Involved in the study	
☒	☐	ChIP-seq
☒	☐	Flow cytometry
☒	☐	MRI-based neuroimaging

Eukaryotic cell lines

Policy information about cell lines

Cell line source(s)	K562, A549, MCF7, HCT116, HepG2, and Hek293T are from American Type Culture Collection (ATCC). H9 cell line replicate 1 is obtained from WiCell and all other replicates are kindly gifted from Lawrence W. STANTON's lab. The HEYA8 cell line was obtained from collaborators. The NCC24 and SNU719 cell lines are from the Korean Cell Line Bank. The IM95 cell line is from Japan Health Science Research Resource Bank.
Authentication	Of all the cell lines, HEYA8, NCC, SNU and IM95 cell lines are authenticated by STR profiling. No authentication is done for K562, A549, MCF7, HCT116 , HepG2, and Hek293T cell lines as they are authenticated by ATCC. No authentication is done for the H9 cell line.
Mycoplasma contamination	All cell lines tested negative for Mycoplasma contamination.
Commonly misidentified lines (See ICLAC register)	None
